# Stratification of the Gut Microbiota Composition Landscape across the Alzheimer's Disease Continuum in a Turkish Cohort

**DOI:** 10.1128/msystems.00004-22

**Published:** 2022-02-08

**Authors:** Süleyman Yıldırım, Özkan Ufuk Nalbantoğlu, Abdulahad Bayraktar, Fatma Betül Ercan, Aycan Gündoğdu, Halil Aziz Velioğlu, Mehmet Fatih Göl, Ayten Ekinci Soylu, Fatma Koç, Ezgi Aslan Gülpınar, Kübra Sogukkanlı Kadak, Muzaffer Arıkan, Adil Mardinoğlu, Mehmet Koçak, Emel Köseoğlu, Lütfü Hanoğlu

**Affiliations:** a Department of Medical Microbiology, Istanbul Medipol University International School of Medicine, Istanbul, Turkey; b Regenerative and Restorative Medicine Research Center (REMER), Research Institute for Health Sciences and Technologies (SABITA), Istanbul Medipol University, Istanbul, Turkey; c Department of Computer Engineering, Erciyes University, Kayseri, Turkey; d Genome and Stem Cell Center (GenKok), Erciyes University, Kayseri, Turkey; e Centre for Host-Microbiome Interactions, Faculty of Dentistry, Oral & Craniofacial Sciences, King's College London, London, United Kingdom; f Graduate Program in Neuroscience, Istanbul Medipol University International School of Medicine, Istanbul, Turkey; g Department of Microbiology and Clinical Microbiology, Erciyes University School of Medicine, Kayseri, Turkey; h Science for Life Laboratory, KTH—Royal Institute of Technology, Stockholm, Sweden; i Department of Preventive Medicine, University of Tennessee Health Science Center, Memphis, Tennessee, USA; j Department of Neurology, School of Medicine, Erciyes University, Kayseri, Turkey; k Department of Neurology, School of Medicine, Istanbul Medipol University, Istanbul, Turkey; University of California San Diego

**Keywords:** gut microbiome, Alzheimer’s disease, 16S rRNA, stratification, brain-gut axis, gut microbiota, precision medicine, precision nutrition

## Abstract

Alzheimer's disease (AD) is a heterogeneous disorder that spans a continuum with multiple phases, including preclinical, mild cognitive impairment, and dementia. Unlike for most other chronic diseases, human studies reporting on AD gut microbiota in the literature are very limited. With the scarcity of approved drugs for AD therapies, the rational and precise modulation of gut microbiota composition using diet and other tools is a promising approach to the management of AD. Such an approach could be personalized if an AD continuum can first be deconstructed into multiple strata based on specific microbiota features by using single or multiomics techniques. However, stratification of AD gut microbiota has not been systematically investigated before, leaving an important research gap for gut microbiota-based therapeutic approaches. Here, we analyze 16S rRNA amplicon sequencing of stool samples from 27 patients with mild cognitive impairment, 47 patients with AD, and 51 nondemented control subjects by using tools compatible with the compositional nature of microbiota. To stratify the AD gut microbiota community, we applied four machine learning techniques, including partitioning around the medoid clustering and fitting a probabilistic Dirichlet mixture model, the latent Dirichlet allocation model, and we performed topological data analysis for population-scale microbiome stratification based on the Mapper algorithm. These four distinct techniques all converge on *Prevotella* and *Bacteroides* stratification of the gut microbiota across the AD continuum, while some methods provided fine-scale resolution in stratifying the community landscape. Finally, we demonstrate that the signature taxa and neuropsychometric parameters together robustly classify the groups. Our results provide a framework for precision nutrition approaches aiming to modulate the AD gut microbiota.

**IMPORTANCE** The prevalence of AD worldwide is estimated to reach 131 million by 2050. Most disease-modifying treatments and drug trials have failed, due partly to the heterogeneous and complex nature of the disease. Recent studies demonstrated that gut dybiosis can influence normal brain function through the so-called “gut-brain axis.” Modulation of the gut microbiota, therefore, has drawn strong interest in the clinic in the management of the disease. However, there is unmet need for microbiota-informed stratification of AD clinical cohorts for intervention studies aiming to modulate the gut microbiota. Our study fills in this gap and draws attention to the need for microbiota stratification as the first step for microbiota-based therapy. We demonstrate that while *Prevotella* and *Bacteroides* clusters are the consensus partitions, the newly developed probabilistic methods can provide fine-scale resolution in partitioning the AD gut microbiome landscape.

## INTRODUCTION

Alzheimer’s disease (AD) is the most common form of dementia worldwide, and its prevalence is estimated to reach 131 million by 2050 (1). AD spans a continuum starting with the nonsymptomatic preclinical stage and advancing through the spectrum of clinical stages. These stages are marked with distinct pathophysiological states (2), namely, the amyloid-tau-neuroinflammation axis. The clinical continuum entails mild memory loss and/or cognitive impairments (mild cognitive impairment [MCI] due to AD) and trajectories for loss of function leading to memory problems besides cognitive impairment (dementia phase), and finally complete loss of independent functioning toward the end stage (3). Moreover, The Alzheimer's dementia phase is further broken down into the stages of mild, moderate, and severe dementia, thereby making AD a complex and highly heterogeneous disease.

Traditionally, the pathogenesis of AD has been attributed to extracellular aggregation of amyloid β-peptides (Aβ) in senile plaques and intracellular depositions of hyperphosphorylated tau that forms neurofibrillary tangles (4). Although numerous clinical trials based on the amyloid postulates have been attempted, virtually all of them have failed (5). The unsettlingly consistent failure of clinical trials targeting single-target amyloid pathways prompted researchers to refine the amyloid hypothesis (6) and even extend it to the periphery (7). Recently, a group of AD researchers asserted that infectious agents reach and remain dormant in the central nervous system (CNS) and undergo reactivation during aging, sparking cascades of inflammation, induction of Aβ, and ultimately neuronal degeneration (8). Chronic inflammation in the CNS mediated by microglial toxicity, as well as systemic inflammation in the periphery, is widely recognized in AD and has been linked to the amyloid cascade hypothesis in animal experiments (9, 10). None of the drugs available today for Alzheimer's dementia slow or stop the damage and destruction of neurons (11). Intervention at different points along the Alzheimer’s continuum should therefore be multimodal and involve targeting neuropathology in brain, systemic inflammation in the body, and metabolic processes in the periphery that escalate the disease in brain (12). Nonpharmacologic, targeted, personalized, and multimodal disease-modifying interventions in AD, including diet and lifestyle changes to optimize metabolic parameters, have recently been under investigation (13–16).

A growing body of evidence suggests that the human gut microbiota is strongly associated with human metabolic processes in all organs, including the brain (17), and is implicated in neuroinflammation via the brain-gut axis (18). Gut microbes across animal models influence the CNS by modulation of neuroimmune function, sensory neuronal signaling, and metabolic activity (19). Several studies using transgenic animal models of AD reported gut microbiota alterations (see reference 19), but these animal models poorly mirror human AD. Unexpectedly, only a few human clinical studies on AD were reported in the literature (20–28). Of these studies, gut microbiota-associated metabolites, such as elevated trimethylamine *N*-oxide (TMAO) in cerebrospinal fluid (CSF) (26) and an altered bile acid profile (28), were directly implicated in AD dementia. Importantly, dietary patterns of AD patients are at the center of precision medicine approaches (29). Disease-modifying approaches involving diet should therefore consider the microbiota in AD. A precision medicine therapy that leverages modulation of gut microbiota could have a beneficial clinical impact on the management of the disease (30). Indeed, a recent study (23) tested the impact of a modified Mediterranean ketogenic diet on gut microbiome composition and demonstrated that the diet can modulate the gut microbiome and metabolites in association with improved AD biomarkers in CSF. These published studies, however, did not comprehensively investigate AD microbiota subtypes (stratification) across the disease continuum prior to the intervention, which can allow personalized therapy. Recently initiated ambitious precision nutrition approaches (31–34) cannot be applied to a highly heterogeneous disease before deconstructing the disease into multiple strata and tailoring therapies accordingly.

In the present study, we postulated that the gut microbiota composition along the AD continuum reflects overlapping gradients of the microbiota community continuum that thus can be stratified into subtypes. We show that while subtypes dominated by *Prevotella* and *Bacteroides* are the consensus partitions, the newly developed probabilistic methods can provide fine-scale stratification of the AD gut microbiome landscape.

## RESULTS

### Study design and participant characteristics.

The study cohort consisted of 47 AD and 27 MCI (all amnestic) patients and 51 subjects who were nondemented controls (*n* = 125). To minimize the dietary confounding effect on the microbiome, we included healthy cohabiting spouses of the patients sharing the same diet as the controls. The control group therefore largely (*n* = 27) comprised partners of the patients. Participants were recruited in two health centers located in different cities. The cohort groups were statistically not different in terms of sex, but age and education factors were significantly different (Table 1) and therefore statistically adjusted in analyses. Expectedly, the groups were also different by cognitive tests, including the Mini-Mental State Exam (MMSE) and the Clinical Dementia Rating (CDR). Most AD participants had very mild or mild dementia, with CDR scores ranging from 0.5 to 3 (median CDR scores of 1 for AD, 0.5 for MCI, and 0 for the control group). The median MMSE scores were significantly higher in the control (score of 27) and MCI (score of 26) groups than the AD group (score of 16). A subset of AD patients (*n* = 12) was clinically asked to undergo lumbar puncture to ascertain diagnosis using CSF biomarkers, including Aβ42/Aβ40 ratio, phosphorylated tau (p-tau), and the p-tau/Aβ42 ratio (see Table S1 in the supplemental material). We collected medication information from the patient’s registry.

**TABLE 1:** Demographic and clinical characteristics of the cohort participants^*a*^

Parameter	Result for group (*n* = 125)		
	Control (*n* = 51)	MCI (*n* = 27)	AD (*n* = 47)
Sex, % (no. female/total)	45 (23/51)	41 (11/27)	49 (23/47)
Age, mean ± SD yr	67 ± 5.3	69.2 ± 6.4	71.4 ± 5.1
Education, mean ± SD yr	7.2 ± 4.1	10.4 ± 5.2	4.4 ± 4.1
MMSE, mean ± SD	27.1 ± 1.7	25.4 ± 2.7	16.9 ± 5.7
CDR, % (no./total)			
0	100	0	0
0.5	0	100 (27/27)	29.8 (14/47)
1	0	0	31.9 (15/47)
2	0	0	29.8 (14/47)
3	0	0	8.5 (4/47)
Aβ1–42/p-tau (pg/mL)	NA	NA	5.97 ± 3.7 (*n* = 14)
Aβ1–42/t-tau (pg/mL)	NA	NA	0.91 ± 0.6 (*n* = 14)
Medication, % (no./total)			
AA	NA	37 (10/27)	27.6 (13/47)
Add	NA	81 (22/27)	87 (41/47)
Adep	NA	66.7 (18/27)	27.6 (13/47)
AE	NA	18.5 (5/27)	8.5 (4/47)
Aht	NA	48 (13/27)	29.8 (14/47)
Apsik	NA	11.1 (3/27)	21.2 (10/47)
Adiab	NA	29.6 (8/27)	19.1 (9/47)
PP	NA	7.4 (2/27)	6.3 (3/47)

a Shown are the demographic characteristics, cognitive performance, concentrations of cerebrospinal fluid biomarkers (Aβ1–42, t-tau, and p-tau), and groups of medicine used by the participants. MCI, mild cognitive impairment group; AD, Alzheimer's disease group; MMSE, Mini-Mental State Exam (MMSE); CDR, Clinical Dementia Rating; AA, antiaggregant; Add, AD treatment; Adep, antidepressant; AE, antiepileptic; Aht, antihypertensive; Apsik, antipsychotic; Adiab, antidiabetic; PP, proton pump inhibitor; NA, not applicable; Aβ1–42, amyloid beta peptide; p-tau, Thr-phosphorylated tau; t-tau, total tau.

10.1128/msystems.00004-22.6 TABLE S1 Levels of cerebrospinal fluid biomarkers of a subset of AD patients. The cutoff values discriminating AD from controls were determined as reported before (69). Download Table S1, DOCX file, 0.02 MB. Copyright © 2022 Yıldırım et al. 2022 Yıldırım et al. https://creativecommons.org/licenses/by/4.0/ This content is distributed under the terms of the Creative Commons Attribution 4.0 International license.

### Microbiome composition is associated with disease status along the AD continuum.

The gut microbiota was profiled using the V3-V4 hypervariable region of the 16S rRNA gene; The Nephele automatic pipeline denoised the paired-end sequences and assigned amplicon sequence variants (ASVs) according to the DADA2 algorithm (35). The Nephele pipeline produced both unrarefied and rarefied ASV tables. The rarefied table included a total of 3,486 ASVs in the table (10,769 sequences/sample) for downstream analyses.

**FIG 1:**
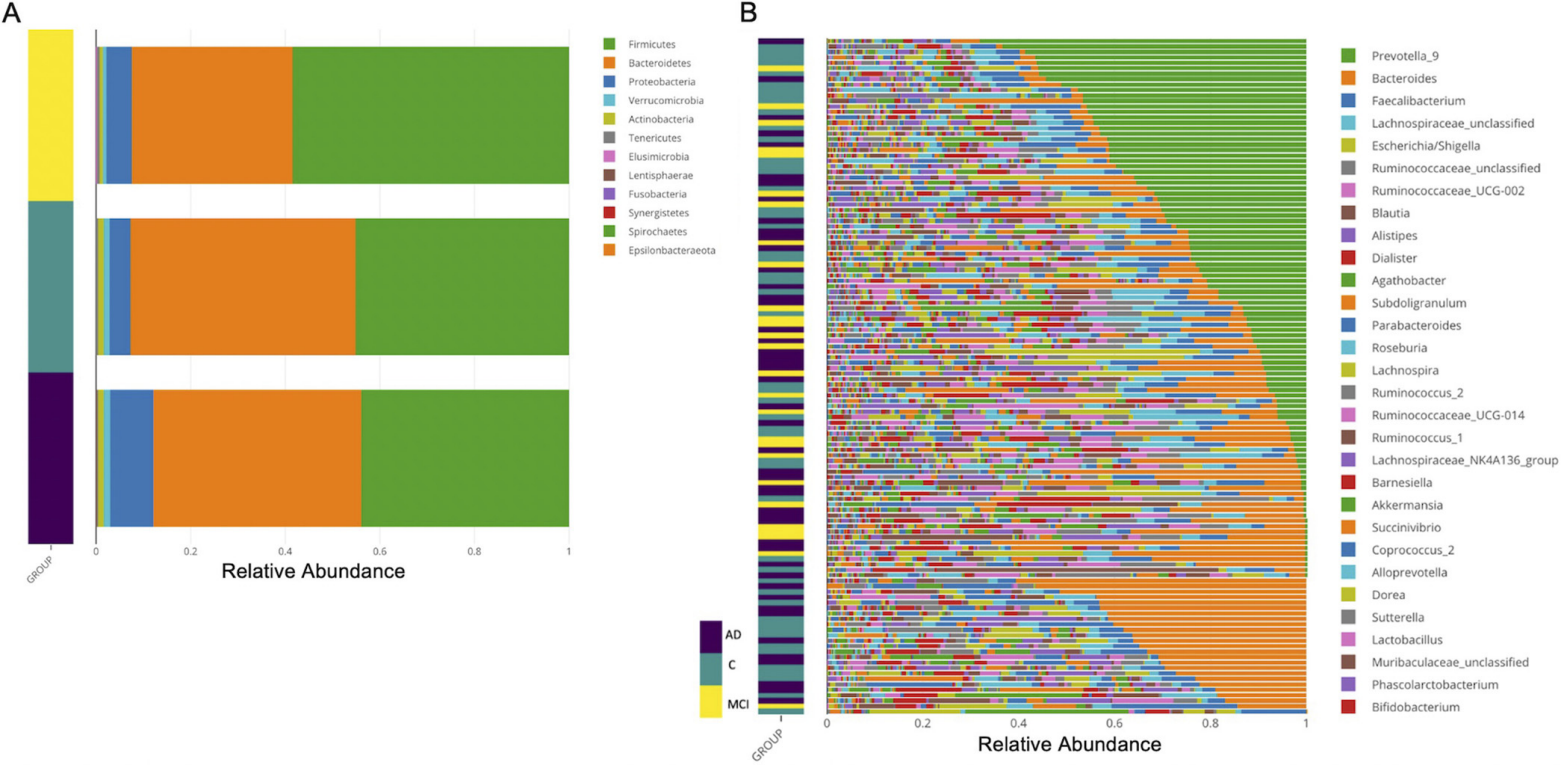
Taxonomic analysis of gut microbiota of the cohort participants. (A) Phylum-level relative abundance in samples from the cohort groups. The phylum-level taxonomic analysis showed a typical human gut microbiota profile in terms of overabundance of *Firmicutes*, *Bacteroidetes*, and *Proteobacteria.* Samples from each group were color coded, as defined to the side. (B) The genus-level relative abundance distributions across samples showed *Prevotella_9* and *Bacteroides* were the most abundant of the top 30 genera across the samples. The color key shows the cohort groups: Alzheimer’s patients (AD) in dark purple, control subjects (C) in teal, and patients with mild cognitive impairment (MCI) in yellow.

We used the resulting rarefied ASV table’s relative abundances to plot taxonomic diversity. The phylum-level taxonomic analysis showed a typical human gut microbiota profile in terms of overabundance of *Firmicutes*, *Bacteroidetes*, and *Proteobacteria* (Fig. 1A). Together with *Verrucomicrobia* and *Actinobacteria*, the five phyla comprised 99% of all reads, but the phylum *Proteobacteria* was overrepresented in AD patient samples. Notably, the genus-level relative abundance distributions across samples showed *Prevotella_9* and *Bacteroides* were the most abundant of the top 30 genera across the samples (Fig. 1B). To perform differential abundance analysis between samples, we sought concordance analysis among multiple tools. ANCOM-BC or ALDEx2, when using covariates in their models, both agreed that only *Ruminoccus_unclassified* is significantly differentially abundant among the groups (data not shown). Nevertheless, when we employed the limma-voom R package (with an age- and sex-adjusted false-discovery rate [FDR] of <0.05) we found that *Prevotella_9*, *Bacteroides*, and members of the *Ruminococcaceae* family were among the top most significantly differentially abundant taxa (ASVs) between the cohort groups (see the differentially abundant ASVs in Table S2, parts A and B, in the supplemental material); whereas these taxa did not reach significance when collapsed at the genus level (Table S2, parts C and D), pointing to the possibility that multiple species of these three taxa exist and may be associated with the health and disease states at the same time. A comprehensive comparative statistical assessment of multivariate and compositional methods (36) demonstrated ALDEx2 or alike tools suffer from low power, while limma-voom and Songbird in their own class were the best performers.

10.1128/msystems.00004-22.7 TABLE S2 Differentially abundant ASV and genus-level taxa between cohort groups as detected by Limma-Voom model (age and sex adjusted). Parts A and B show a list of differentially abundant ASVs between AD versus C and MCI versus C, respectively. Parts C and D show differentially abundant genus-level taxa between AD versus C and MCI versus C, respectively. Download Table S2, XLSX file, 0.02 MB. Copyright © 2022 Yıldırım et al. 2022 Yıldırım et al. https://creativecommons.org/licenses/by/4.0/ This content is distributed under the terms of the Creative Commons Attribution 4.0 International license.

Alpha diversity indices (Shannon and inverse Simpson) (see Fig. S1A to D in the supplemental material) did not show significant differences after multiple testing corrections (Kruskal-Wallis, FDR > 0.05), but only the richness index, Chao1, showed a significant difference between MCI and the control group (pairwise Wilcoxon rank sum test, *P* = 0.008074).

10.1128/msystems.00004-22.1 FIG S1 Alpha diversity analysis. Alpha diversity indices were calculated using a rarefied ASV table as input to the phyloseq R package. Violin plots show (A) Chao1 index, (B) inverse Simpson, (C) observed species, and (D) Shannon diversity index. The Shannon and inverse Simpson results did not show significant differences after multiple testing corrections (Kruskal-Wallis, FDR > 0.05), but the richness index, Chao1, showed significant differences between MCI and the control group (pairwise Wilcoxon rank sum test, *P* = 0.008074). Download FIG S1, PDF file, 0.2 MB. Copyright © 2022 Yıldırım et al. 2022 Yıldırım et al. https://creativecommons.org/licenses/by/4.0/ This content is distributed under the terms of the Creative Commons Attribution 4.0 International license.

**FIG 2:**
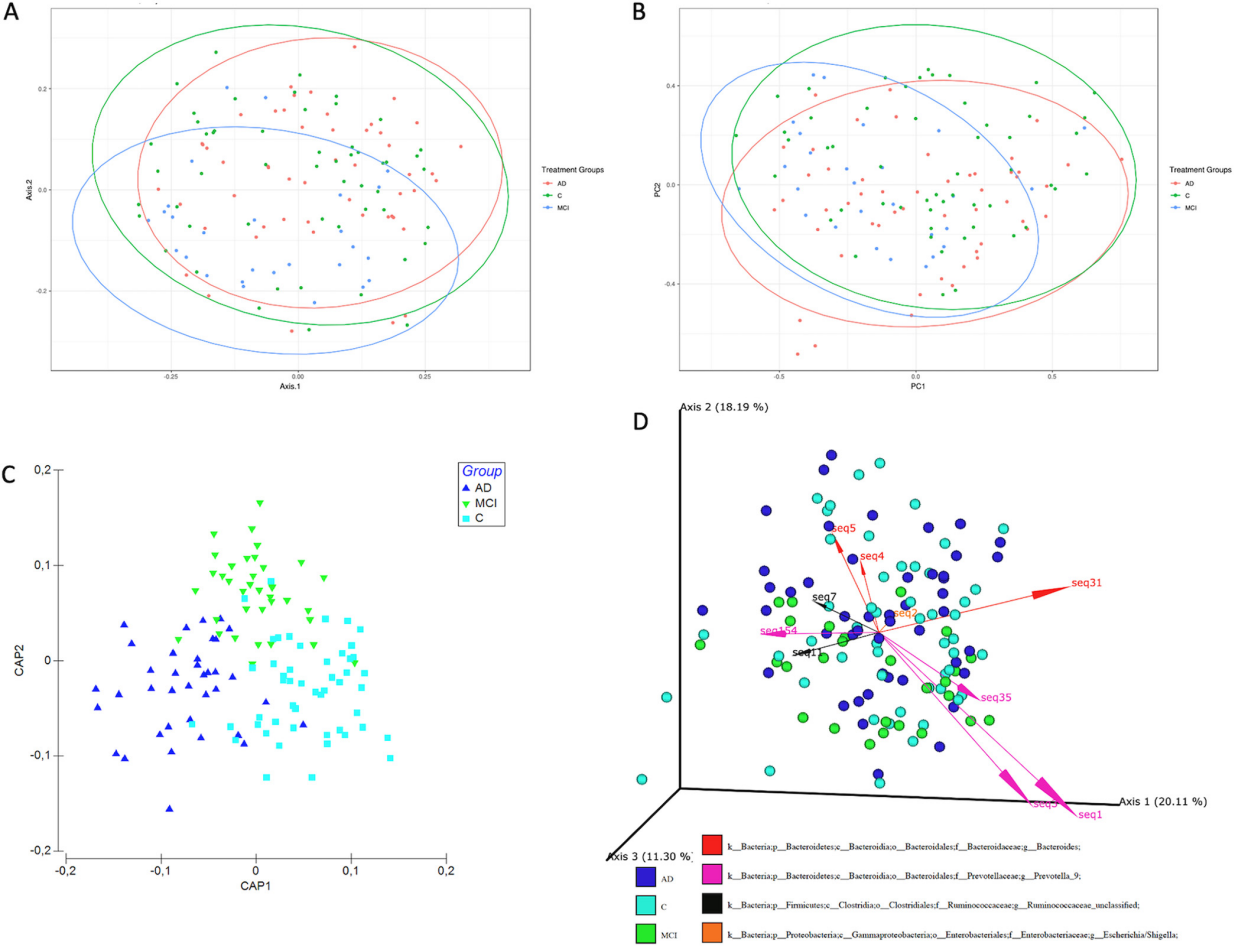
Beta diversity analysis of stool samples shows clear separation of the groups. Shown is a principal-coordinate analysis (PCoA) plot of the gut microbiome based on (A) Bray-Curtis distances between the samples and (B) Jaccard distances between the samples. Ellipses represent 95% confidence intervals. (C) Canonical analysis of principal coordinates (CAP) ordination of the samples. (D) Robust Aitchison principal-coordinate analysis using DEICODE. Arrows represent individual taxa driving the separation of samples along the principal components, and their length correlates with the feature loadings and the biplot axis. Colored dots represent individual subjects and are colored according to the designated cohort group.

We employed both relative abundance-based and recently developed compositionally aware tools, namely, DEICODE (37) and Songbird (38), to compare the composition and structure of bacterial communities in samples using multiple beta diversity indices (Bray-Curtis, Jaccard, and Aitchison). The principal-coordinate analysis (PCoA) showed separation of the three groups by both Bray-Curtis and Jaccard indices (Fig. 2A and B). We used the adonis2 function in the qiime2 plugin (q2-diversity) to perform permutational multivariate analysis of variance (PERMANOVA) with 999 permutations, and the included interaction terms (see Table S3 in the supplemental material) and separation of the groups were highly significant (*P* = 0.0001). Age and sex also significantly contributed to the total variance (*P* < 0.001), but the interaction terms were not significant. Furthermore, tests of dispersion between groups (PERMDISP) indicated only the dispersion MCI group is significantly heterogenous (pairwise comparisons, *P* = 0.033 for AD versus MCI, *P* = 0.024 for control versus MCI, and *P* = 0.672 for AD versus C), which may be attributed to unbalanced design. We added further support for the separation of the three groups representing the AD continuum from other ordinations. The canonical analysis of principal-coordinates (CAP) analysis unambiguously showed the three groups are distinct (trace statistic = 0.86855 and *P* = 0.001 for 999 permutations) (Fig. 2C). The final support in beta diversity was provided by the DEICODE analysis (robust Aitchison principal-component analysis [PCA]) (PERMANOVA, *P* = 0.02) (Fig. 2D), which indicated that the three groups are distinct, and the community clusters are largely driven by a subset of ASVs with the taxonomic assignments *Prevotella_9*, *Bacteroides*, an unclassified genus within *Ruminococcaceae* family (*Ruminococcaceae_unclassified*), and Escherichia/*Shigella*. Moreover, the co-occurrence analysis using SparCC showed that *Prevotella_9* and *Bacteroides* were negatively correlated (correlation = −0.4445, FDR = 0.09355). Finally, the genus-level PCoAs showed partially overlapping clusters of these two taxa, while the groups overall were also significantly separated (PERMANOVA, *P* < 0.0001) (see Fig. S2A to C in the supplemental material). Collectively, the differential abundance and beta diversity analyses showed gut microbiota composition is associated with the disease status along the AD continuum. We therefore paid particular attention to these two taxa in the rest of the downstream analyses.

10.1128/msystems.00004-22.2 FIG S2 Multidimensional scale (MDS) analysis of genus relative abundances show gradient of *Prevotella_9* and *Bacteroides*. (A) MDS analysis of 16S rRNA genus-level abundances. (B) Gradient of *Prevotella_9* abundances across the samples. (C) Gradient of *Bacteroides* abundances across the samples. Download FIG S2, PDF file, 0.2 MB. Copyright © 2022 Yıldırım et al. 2022 Yıldırım et al. https://creativecommons.org/licenses/by/4.0/ This content is distributed under the terms of the Creative Commons Attribution 4.0 International license.

10.1128/msystems.00004-22.8 TABLE S3 PERMANOVA of covariates using the adonis2 function in the qiime2 diversity plugin. Download Table S3, DOCX file, 0.02 MB. Copyright © 2022 Yıldırım et al. 2022 Yıldırım et al. https://creativecommons.org/licenses/by/4.0/ This content is distributed under the terms of the Creative Commons Attribution 4.0 International license.

**FIG 3:**
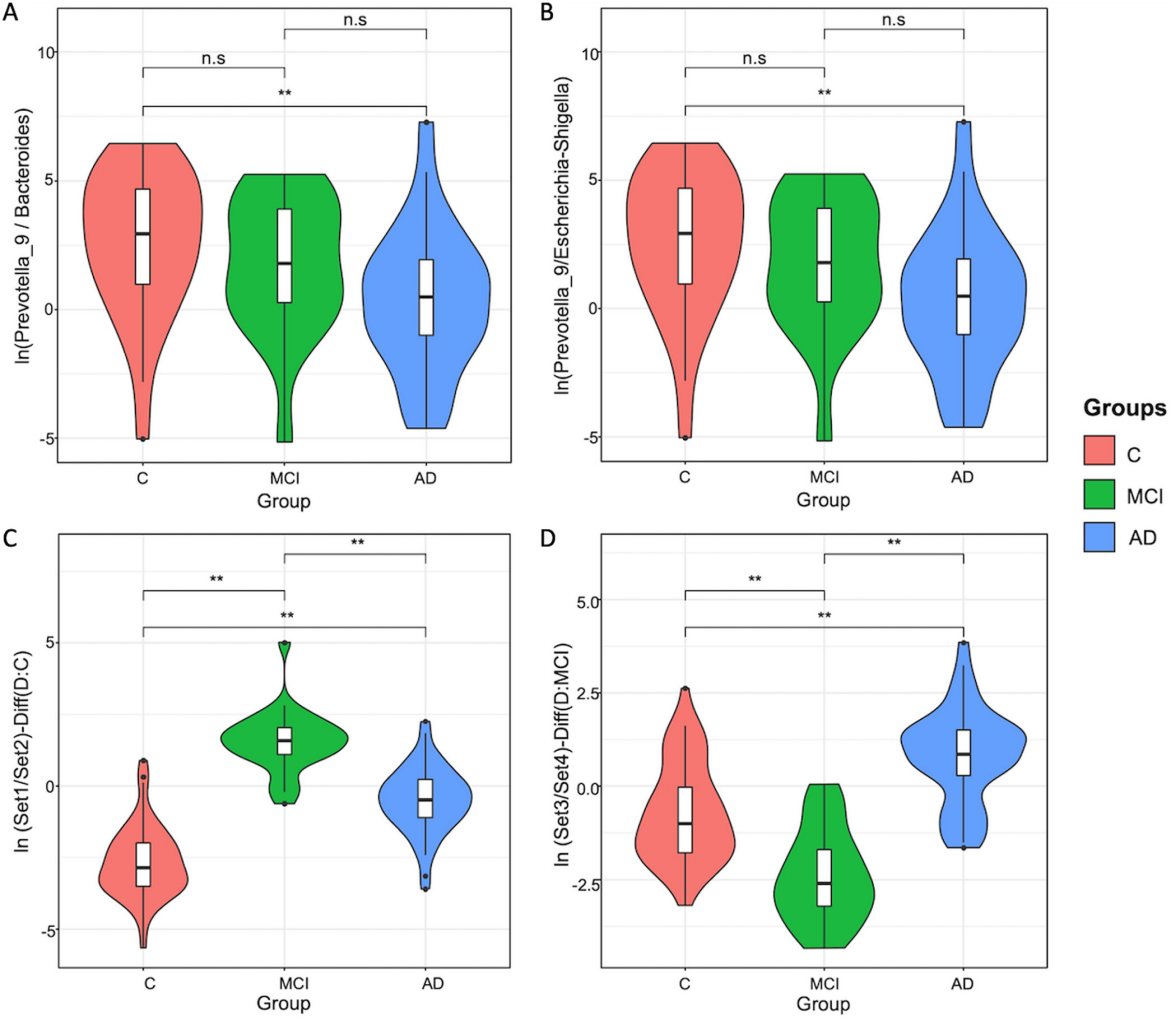
Enrichment analysis by multinomial regression derived from Songbird. (A) Violin plots of the log ratios of the taxa *Prevotella_9* and *Bacteroides* and (B) violin plots of the log ratios of the taxa *Prevotella_9* and Escherichia/*Shigella.* Both ratios in panels A and B significantly separate the AD group from the control group, but not from the MCI group (Welch’s *t* test, FDR-adjusted *P* = 0.04). (C) Violin plots of the log ratios of the taxon sets (ratio of set 1 to set 2, representing the top and bottom 25%, respectively) and (D) violin plots of the log ratios of the taxon sets (ratio of set 3 to set 4, representing the top and bottom 25%, respectively) significantly separate the cohort groups (Welch’s *t* test, FDR-adjusted *P* = 0.0002).

Enrichment analysis by multinomial regression embedded in the Songbird tool with regard to covariates (coded as formula: Age+Sex+Edu+MMSE+CDR+Groups(levels=(“C”, “MCI”, “AD”)), indicated that the natural log ratios of *Prevotella_9* to *Bacteroides* and *Prevotella_9* to Escherichia/*Shigella* significantly separated the AD group from the control group (Welch’s *t* test, FDR-adjusted *P* = 0.04), but not from the MCI group (Fig. 3A to D). (Note that the formula follows the Patsy formatting, as described in more detail in Materials and Methods.) Importantly, the Songbird tool excluded 25 samples from this analysis due to zero-rich abundances that do not allow for center-log ratio calculations. We therefore tested the natural log ratio of the top 25%, allowing us to include all samples in the analysis to the bottom 25% (sets 1 and 2 in Table S4, part A, in the supplemental material) of the ranked ASVs associated with the AD relative to the control group; also, the same ratios for the MCI group relative to the control group (sets 3 and 4 in Table S4, part B) and the ASVs enriched in each group were visualized with Qurro (39). Both sets of ranked log ratios revealed significant differences between the log ratios of features differentiating groups (Welch’s *t* test, FDR-adjusted *P* = 0.0002).

10.1128/msystems.00004-22.9 TABLE S4 Taxon names from the enrichment analysis as determined by multinomial regression embedded in Songbird. Sets 1 and 2 are listed in part A, and sets 3 and 4 are listed in part B. Download Table S4, XLSX file, 0.09 MB. Copyright © 2022 Yıldırım et al. 2022 Yıldırım et al. https://creativecommons.org/licenses/by/4.0/ This content is distributed under the terms of the Creative Commons Attribution 4.0 International license.

### Discrete multiple subsets of gut microbiota exist along the AD continuum.

Considering the preceding results, we postulated that the gut microbiota profile along the AD continuum does not represent a single state, but rather distinct yet overlapping community types. We addressed this hypothesis using four unique methods: (i) partitioning around medoid (PAM)-based clustering (40), (ii) fitting Dirichlet multinomial mixture (DMM) models to partition microbial community profiles into a finite number of clusters (41) using the Laplace approximation, (iii) fitting latent Dirichlet allocation (LDA) (42, 43) using the perplexity measure, and (iv) analyzing topological futures of data density (44) based on the Mapper algorithm to capture subtle and nonlinear patterns of high-dimensional data sets and population-level stratification.

**FIG 4:**
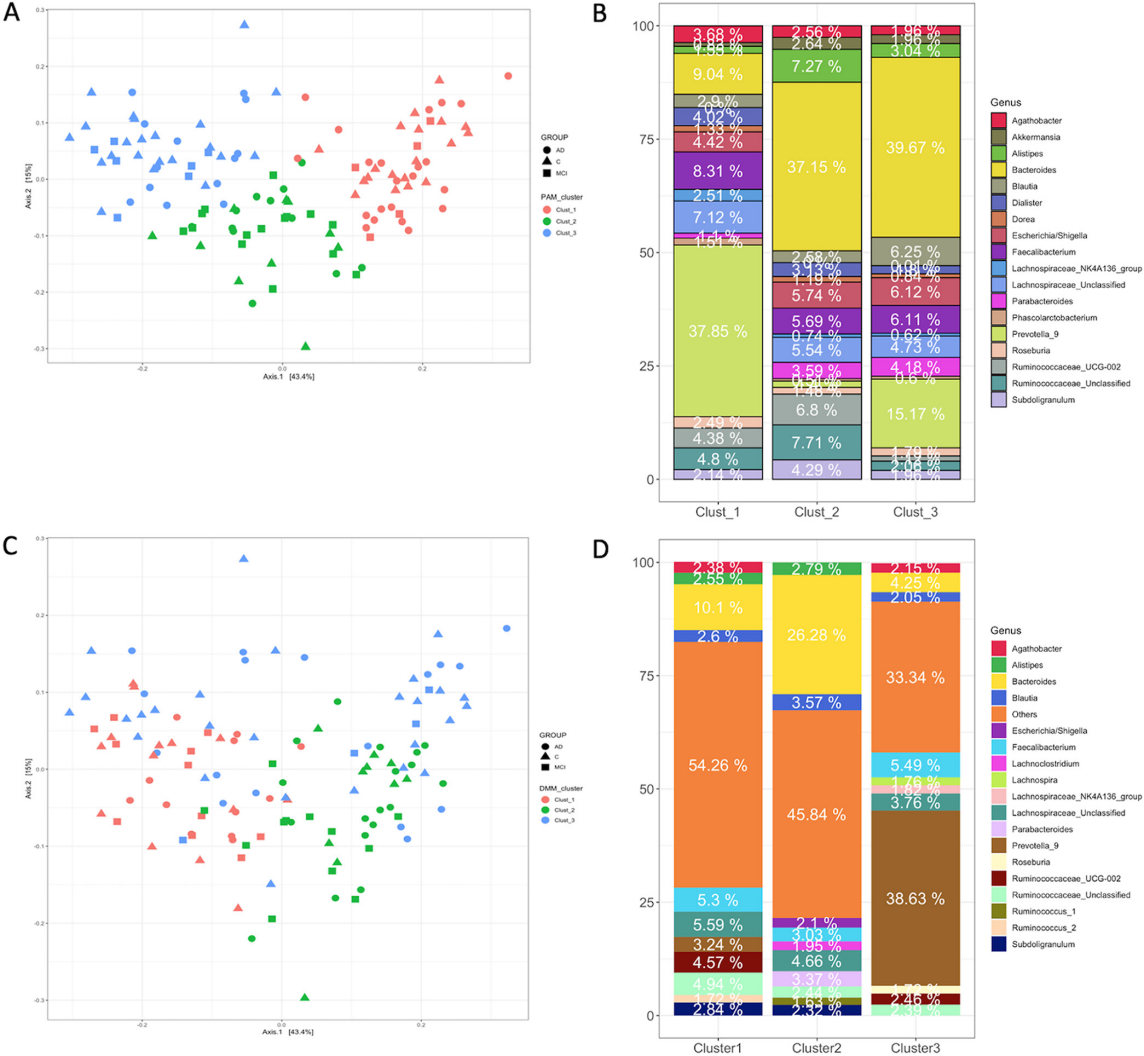
Stratification of the gut microbiota composition using PAM-based clustering (top) and DMM (bottom). (A) NMDS ordination of samples within each cluster using relative abundances. (B) Relative abundances of taxa in each PAM cluster. (C) Nonmetric multidimensional scaling (NMDS) ordination of samples within each DMM cluster. (D) Relative abundances of taxa in each DMM cluster. Both PAM clustering and DMM indicate three clusters (clusters 1, 2, and 3). The genus *Bacteroides* was the most abundant taxon in the first two clusters, and the third cluster was dominated by *Prevotella_9.* Note the highly enriched taxon *Bacteroides* in cluster 2 and decreased *Faecalibacterium* abundance contrasting with the elevated abundance of Escherichia/*Shigella*.

The PAM-based clustering identified three (*k* = 3) distinct clusters based on Gap statistics (see Fig. S3A in the supplemental material). PCoA of the sample abundances in the three clusters indicated significant separation of the clusters (PERMANOVA, *P* = 0.001) (Fig. 4). We confirmed the optimum number of clusters using both Jensen-Shannon and Bray-Curtis distance metrics (data not shown). The relative abundance of the genus *Prevotella_9* dominated cluster 1, while the genus *Bacteroides* showed the highest relative abundances in the other two clusters (Fig. 4B).

10.1128/msystems.00004-22.3 FIG S3 Determining the number of clusters in the gut microbiota. Shown are the optimal number of clusters based on (A) the Gap statistic with standard error bars for PAM analysis and (B) the Laplace method for evaluating model fit for increasing number of Dirichlet mixture components. Both Gap statistics and Laplace method show 3 clusters as optimum. Download FIG S3, PDF file, 0.1 MB. Copyright © 2022 Yıldırım et al. 2022 Yıldırım et al. https://creativecommons.org/licenses/by/4.0/ This content is distributed under the terms of the Creative Commons Attribution 4.0 International license.

Next, we employed Dirichlet multinomial mixture probabilistic community modeling using the DirichletMultinomial R package (41) and fitting genus-level absolute abundances. Based on Laplace approximation, three clusters (clusters 1, 2, and 3) represented the best model fit (Fig. 3B), which was congruent with the PAM-based clustering. The PCoA of these clusters and PERMANOVA pairwise tests further supported the existence of three distinct clusters within the microbial community (PERMANOVA, *P* = 0.01) (Fig. 4C). The genus *Bacteroides* was the most abundant taxon in the first two clusters, and the third cluster was dominated by *Prevotella_9* (Fig. 4D). Notably, cluster 2 included a significantly higher abundance of *Bacteroides* (26.3%) than cluster 1 (9.9%) and cluster 3 (4.7%). In addition to highly enriched *Bacteroides* in cluster 2, the decreasing trend of *Faecalibacterium* abundance and elevated abundance of inflammation associated with *Escherchia*/*Shigella* suggested that cluster 2 can be named “*Bacteroides2* (*Bact2*) *enterotype*,” as recently described (45, 46). Reportedly, the abundance of *Bacteroides* in Bact2 enterotype can reach as high as 78% in patients with inflammatory bowel disease and is associated with systemic inflammation. These results suggest that cluster 2 includes patients with aggravated systemic inflammation.

**FIG 5:**
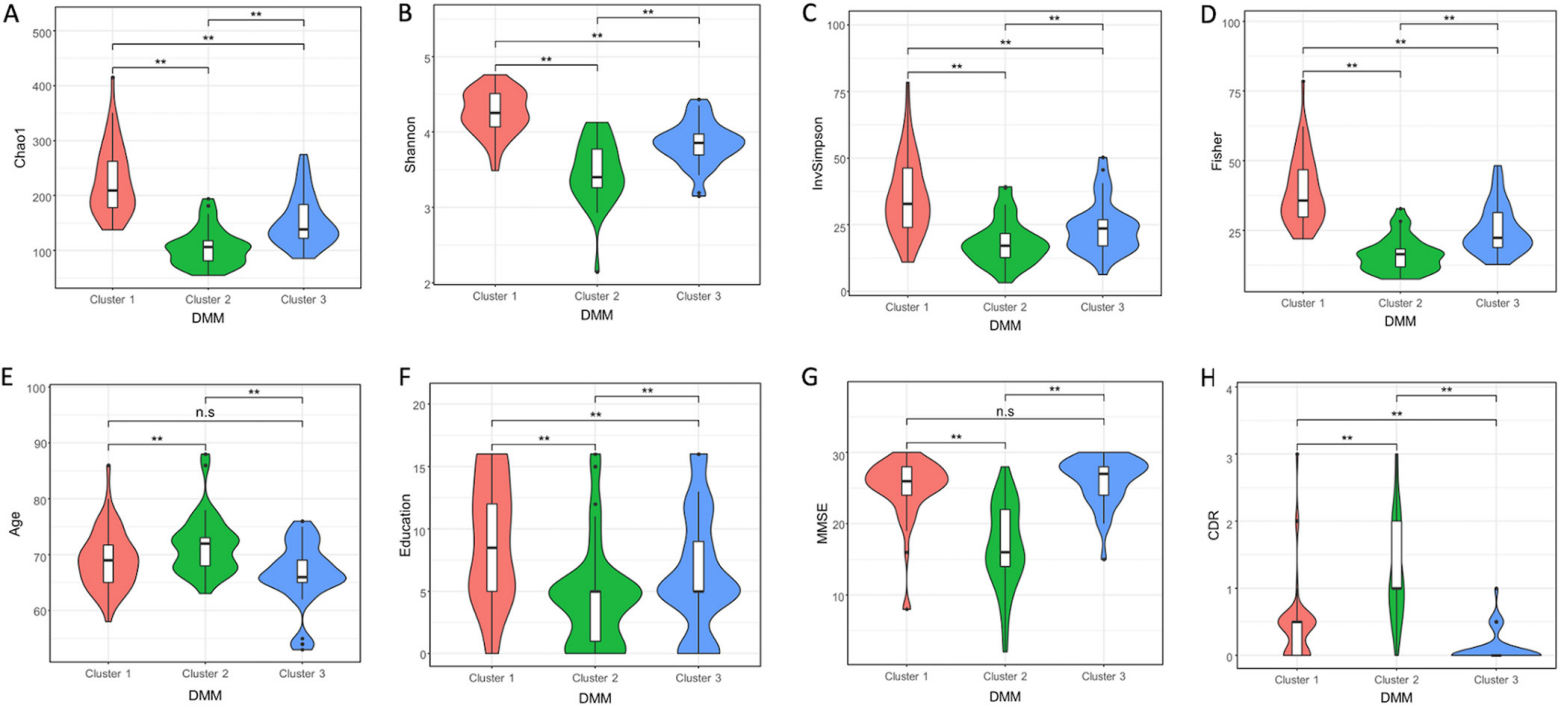
Clinical metadata and diversity within DMM clusters are significantly different. (A to D) Violin plots show (A) the Chao1 index among the DMM clusters, (B) the Shannon index, (C) the inverse Simpson (InvSimpson) index, and (D) the Fisher index. (E) Age distribution across the DMM clusters. (F) Level of Education across DMM clusters. (H) MMSE box plots and (I) CDR box plots across the clusters. The alpha diversity indices (Chao1, Shannon, and inverse Simpson) were statistically different between all three clusters after Benjamini-Hochberg FDR adjustment. However, differences in CDR, MMSE, age, sex, and education were not significant between the clusters (Kruskal-Wallis test followed by Dunn’s *post hoc* test, FDR of <0.05).

We also performed SIMPER analysis based on Bray-Curtis distance to identify taxa contributing most to dissimilarities between clusters (data not shown). *Bacteroides*, *Prevotella_9*, *Faecalibacterium*, and taxa within *Ruminococcaceae* family ranked among the top 10 taxa contributing the most to differences between the three DMM clusters. To examine which factors were associated with the DMM clusters, we analyzed the distribution of clinical metadata and diversity metrics within the clusters. Alpha diversity indices (Chao1, Shannon, and inverse Simpson) were statistically different between all three clusters after Benjamini-Hochberg FDR adjustment. In addition, the clinical factors (CDR, MMSE, age, sex, and education) were also all significantly different among the clusters (Fig. 5A to H), except age and MMSE, for which only cluster 2 reached significance (Kruskal-Wallis test followed by Dunn’s *post hoc* test, FDR < 0.05). (Fisher’s exact test was used for the parameter sex, which is not shown in the figure.)

**FIG 6:**
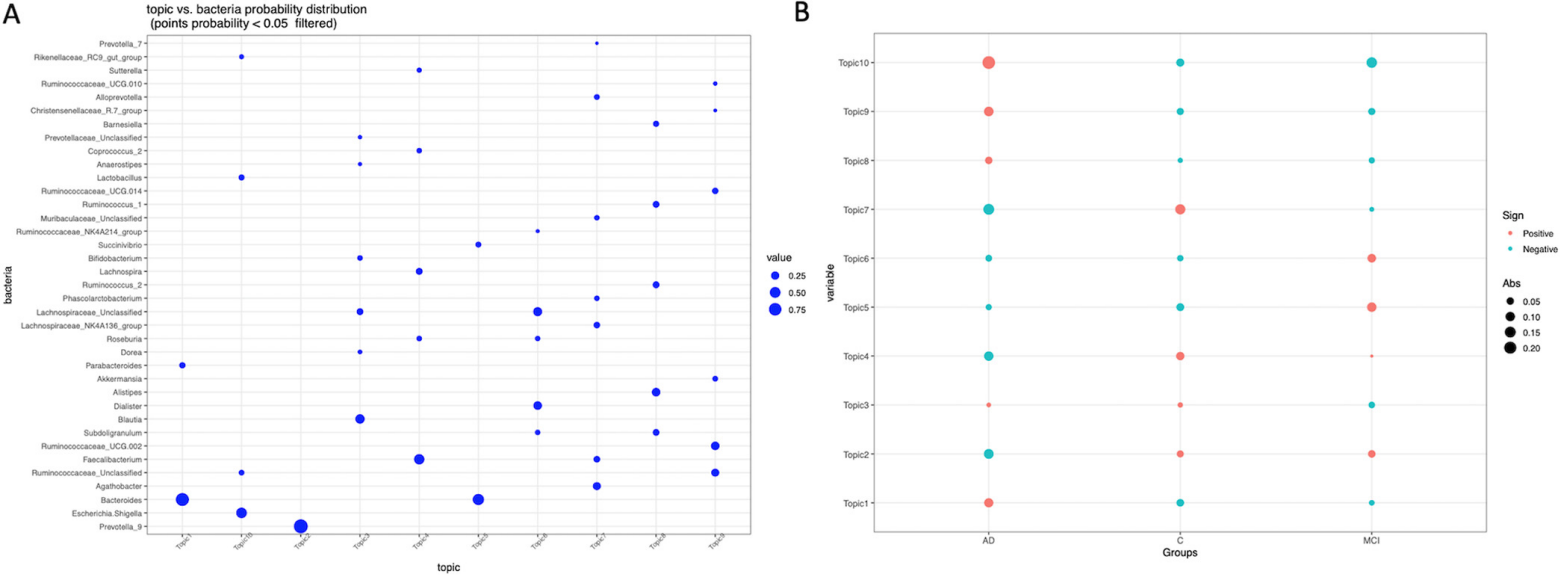
Gut microbiota stratification using LDA model. (A) The LDA model’s perplexity parameter and log-likelihood values to find the optimal number of clusters. Of the 10 subgroups two subgroups were dominated by *Bacteroides* (topics 1 and 5), as well as a subgroup (topic 2) dominated by *Prevotella_9*, with 97% probability. One subgroup (topic 10) was highly enriched by the genus Escherichia/*Shigella* and also included putatively opportunistic bacteria, such as *Enterococcus* and Klebsiella, while subgroups 4, 6, and 9 were dominated by putative butyrate and acetate producers or mucinophilic bacteria. (B) Associations between subgroups and the cohort groups deduced by the Quetelet index, estimating the relative change of the frequency of occurrence of a latent subgroup among all the samples compared to that among the samples of the cohort groups. The red dots indicate positively associated subgroups (topics) with the cohort groups.

As a third method, we tested the LDA potential to stratify gut microbiota of the cohort participants. This unsupervised machine learning technique is increasingly finding acceptance in the field of microbiome studies (47–49) for its unique ability to reveal latent or hidden groups within the data cloud. Figure S4 in the supplemental material shows the LDA model’s perplexity parameter and log-likelihood values to find the optimal number of clusters. Both parameters continued to partition the community without reaching a clear optimum; we therefore presented the first 10 subgroups, also to allow for comparison with the results of topological analysis. The perplexity parameter not reaching a clear optimum is unexpectedly consistent with recent publications using LDA in microbial ecology (47–49). Bacterial probability distributions (ranked by a probability of ≥1% in descending order) across the subgroups are displayed in Fig. 6A. Interestingly, of the 10 subgroups, two subgroups were dominated by *Bacteroides* (topics 1 and 5), and a subgroup (topic 2) was dominated by *Prevotella_9*, with 97% probability. These subgroups therefore resemble subgroups detected by PAM and DMM in terms of prevalence of *Bacteroides* and *Prevotella_9*. Unlike DMM and PAM, however, LDA detected a distinct subgroup (topic 10) with the top-ranking genus Escherichia/*Shigella*, which also included putatively opportunistic bacteria, such as *Enterococcus* and Klebsiella. Subgroups 4, 6, and 9 were conspicuous with the genera known to produce butyrate and acetate or were mucinophilic. Even though we present the first 10 subgroups (topics) here, we also examined higher-order subgroups and observed that the 10 subgroups are further partitioned into additional subgroups, such as subgroups in which the top-ranking probability of *Lactobacillus* and *Akkermansia* emerges. Finally, we plotted the Quetelet index by subgroups to infer associations between subgroups and the cohort groups (Fig. 6B). The Quetelet index estimates the relative change of the occurrence frequency of a latent subgroup among all the samples compared to that among the samples of the cohort groups. The index showed subgroups 1, 8, 9, and 10 are positively associated with the AD group. The subgroup 9 is enriched by the members of *Ruminococcaceae* family. The top-ranking *Ruminococcaceae_UCG_002* and *Akkermansia* are more abundant in the AD group than the control group according to limma-voom analysis. *Akkermansia* overabundance in AD gut microbiota is counterintuitive but was previously reported by others (25), and this genus is more abundant in the gut microbiota of Parkinson’s patients as well (50). Subgroup 10, where Escherichia/*Shigella* is the top-ranking genus, is strongly associated with the AD group but negatively associated with other groups. Conversely, subgroups 2, 4, and 7, which are enriched by short-chain fatty acid (SCFA) producers, are positively associated with the control and MCI groups but negatively associated with AD.

10.1128/msystems.00004-22.4 FIG S4 Latent Dirichlet allocation model performance. The LDA model’s perplexity parameter (top) and log-likelihood values (bottom) do not show a clear optimum point. Download FIG S4, PDF file, 0.2 MB. Copyright © 2022 Yıldırım et al. 2022 Yıldırım et al. https://creativecommons.org/licenses/by/4.0/ This content is distributed under the terms of the Creative Commons Attribution 4.0 International license.

**FIG 7:**
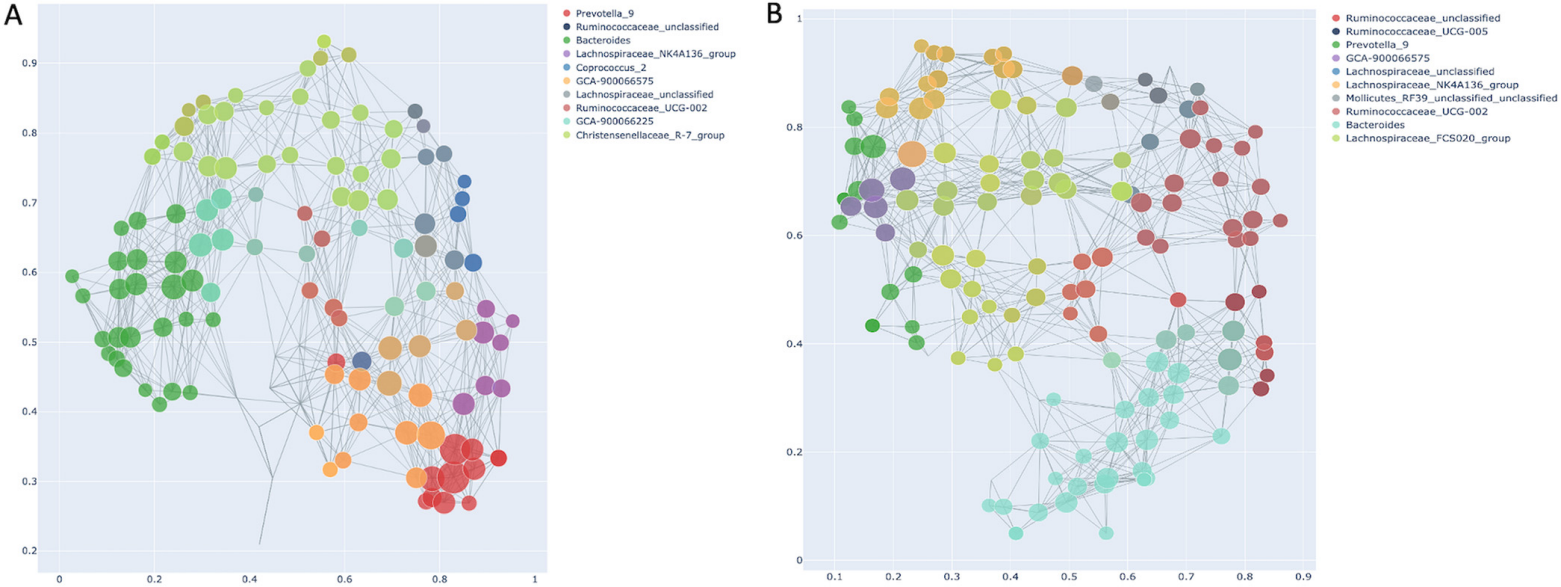
Stratification of gut microbiota using topological data analysis (TDA) based on SAFE scores of enriched taxa. The TDA enrichment network shows the top 10 driver taxa with the highest SAFE scores. The legend shows the top 10 taxa ranked by SAFE scores. Node color is based on the corresponding taxon. Marker size is scaled according to the SAFE enriched score of the taxon. A node in the network represents a group of samples with similar microbiome profiles, and if common samples between nodes are shared, then the nodes are linked. To construct the TDA network first dimension reduction (*filtering* function) was applied using PCoA, and to check distance-based variations, TDA was constructed using two commonly used distance metrics. (A) TDA network showing top 10 driver taxa with the highest SAFE scores and with the Bray-Curtis distance metric used at the filtering step. (B) TDA network showing top 10 driver taxa with highest SAFE scores and with the Jensen-Shannon distance metric used at the filtering step. Both networks indicate largely similar stratification profiles of the top 10 driver taxa and include *Prevotella_9*, *Bacteroides*, *Rumunococaceae_unclassified*, species of *Lachnospiraceae*, and *GCA90006675*, while *Caprococcus_2* and *Mollicutes_RF39_unclassified* were not shared by the two networks.

Another and final method we employed to stratify gut microbiota was topological data analysis (TDA), based on the Mapper algorithm (51) embedded in the recently developed tmap tool (44). The tmap tool was developed for network representation for stratification and association study of high-dimensional microbiome data. After constructing TDA microbiome network using the Mapper algorithm (ordination, covering, and DBSCAN clustering), the workflow in the second step includes computation of a modified version of the spatial analysis of functional enrichment (SAFE) scores to map both the metadata and microbiome features into the TDA network to generate their vectors of SAFE scores. Vectors of SAFE scores are then used to perform ranking and ordination, as well as coenrichment relations to delineate relationship between metadata and microbiome features. To construct the TDA network, we first applied dimension reduction (filtering) in PCA using Bray-Curtis distance, followed by the above algorithm, and also repeated the entire analysis using Jensen-Shannon distance to reveal the effect of the distance metric, if any. To understand how driver taxa relate to each other and to the clinical metadata, we performed principal-component analysis (PCA) of SAFE scores. Figure 7A shows the TDA network and PCA (Bray-Curtis distance) of the taxa metadata based on SAFE scores (see Data Set S1, tab 1, in the supplemental material), respectively. We obtained a similar TDA network profile using Jensen-Shannon distance (Fig. 7B) and SAFE scores (Data Set S1, tab 2). The size of each marker is scaled according to its SAFE score, and only the top 30 bacterial species are shown in PCA figures for clarity. A node in the network represents a group of samples sharing similar bacterial genus profiles. Two given nodes are linked when common samples are shared between the two nodes. The TDA analysis using both distance indices resulted in very similar stratification profiles with the top 10 SAFE-scoring genera, including *Prevotella_9*, *Bacteroides*, *Rumunococaceae_unclassified*, species of *Lachnospiraceae*, and *GCA90006675.* Unsurprisingly, a few taxon rankings differed between the two profiles, such as *Caprococcus_2* and *Mollicutes_RF39_unclassified*.

10.1128/msystems.00004-22.10 DATA SET S1 (Tab 1) Ranking of SAFE scores calculated using the “filter” function based on Bray-Curtis distance. (Tab 2) Ranking of SAFE scores calculated using the “filter” function based on Jensen-Shannon distance. Download Data Set S1, XLSX file, 0.05 MB. Copyright © 2022 Yıldırım et al. 2022 Yıldırım et al. https://creativecommons.org/licenses/by/4.0/ This content is distributed under the terms of the Creative Commons Attribution 4.0 International license.

**FIG 8:**
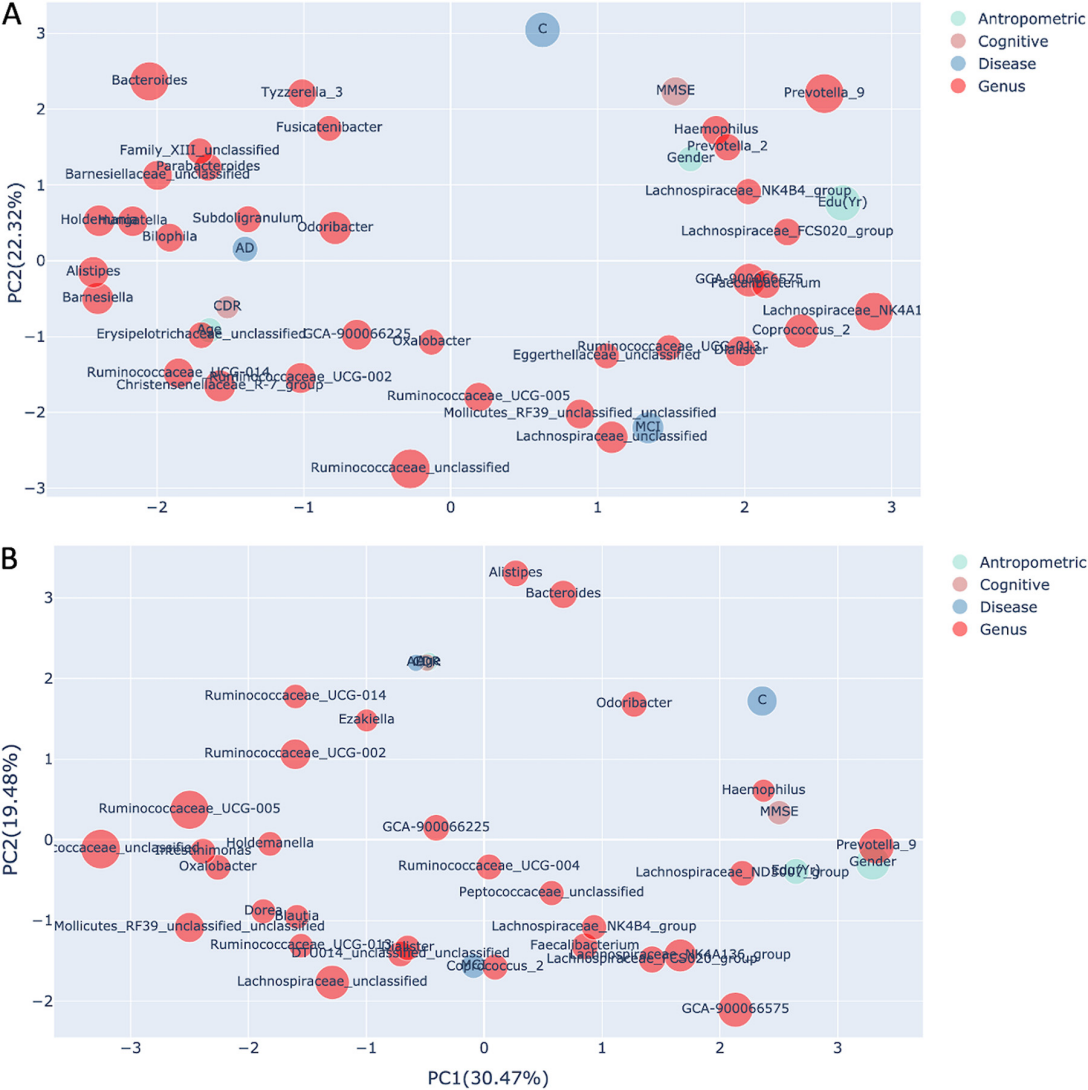
Principal-component analysis of SAFE scores of taxa and clinical variables shows the overall pattern of the association. (A) Principal-component analysis of the SAFE scores and clinical metadata based on Bray-Curtis distance showing the associations of taxa (red [only the top 30 are shown]) with the clinical variable category (blue, brown, or green). Marker size is scaled with respect to taxon SAFE scores. Samples from the control group are shown to be associated with the taxa *Prevotella_9*, species of *Lachnospiraceae*, and *GCA90006675* and the clinical variables, gender, MMSE, and education, while AD samples were more closely associated with taxa such as *Subdoligranulum*, *Odoribacter*, *Bilophila*, *Alistipes*, and *Bacteroides* and CDR and age. The MCI group was coenriched with *Ruminocoaceae_unclassified*, some members of the *Lachnospiraceae* family, *Faecalibacterium*, *Ruminocoaceae_UCG_005*, and *Lachnospiraceae_unclassified.* (B) Principal-component analysis of the SAFE scores of taxa (red [only the top 30 are shown]) and clinical variable category based on Jensen-Shannon distance. The associations of taxa and clinical variables with the cohort groups are largely coherent with the PCA based on Bray-Curtis distance.

Furthermore, Fig. 8A and B show taxa and host covariates based on Bray-Curtis and Jensen-Shannon distances, respectively. Regardless of the distance metric, all three groups were clearly separated. The drivers of microbiome stratification (*Prevotella_9*, *Bacteroides*, and *Ruminococcus_unclassified*) are placed near the control, AD, and MCI groups, respectively, in both PCA figures. Of the clinical metadata, MMSE, sex, and education were grouped with the control group and coenriched with *Prevotella_9*, but also with the *Prevotella_2*, Haemophilus, and *Lachnospiraceae_NK4B4* group. Conversely, CDR, age, and the AD group were clustered together and coenriched with taxa such as *Subdoligranulum*, *Odoribacter*, *Bilophila*, and *Alistipes.* The MCI group was coenriched with *Ruminocoaceae_unclassified*, *Mollicutes_RF39_unclassified*, *Ruminocoaceae_UCG_005*, and *Lachnospiraceae_unclassified.* However, some taxa, such as *Odoribacter*, were placed near the control group in the Jensen-Shannon distance PCA (Fig 8B), suggesting coenrichment of certain taxa can be somewhat influenced by the preferred distance metric.

### Identification of signature taxa for AD continuum and association with metadata.

**FIG 9:**
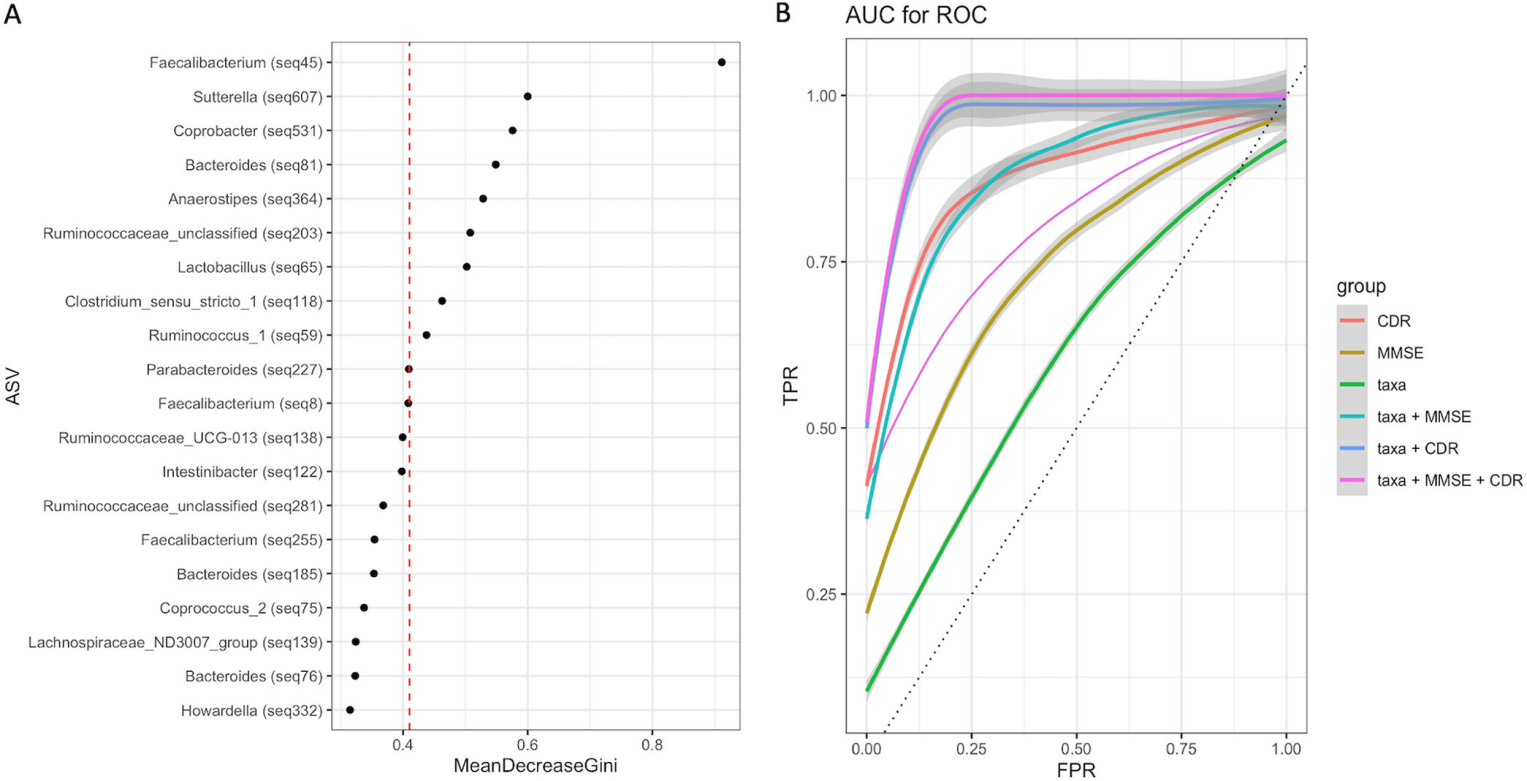
Random forest (RF) model of selected features of gut microbiota and psychometric test scores (MMSE and CDR). (A) ASVs with a mean decrease in Gini values above the breakpoint curve were chosen to be part of the classifier. ASV45, classified as *Faecalibacterium*, showed the highest importance in the model. (B) Average ROC curves with confidence intervals and AUROC obtained by the random forest models for predicting disease status. The ROC value for these selected nine taxa alone showed moderate accuracy (AUROC, 63%). When MMSE and/or CDR and the nine taxa are considered groupwise, the AUROC ranged from 0.74 to 1.0.

To identify signature taxa, we constructed a random forest (RF) model based on selected features of gut microbiota and psychometric test scores (MMSE and CDR) that are typically used as a proxy in clinical diagnosis. We first used Songbird to select 300 ASV features (the top 25%) that differentiate between the healthy (control) group and the disease groups (MCI and AD). We then plotted the ASVs with the first 20 highest mean decrease in Gini values (Fig. 9A) and included ASVs with mean decrease in Gini values above the breakpoint curve in the RF analysis. We identified the following 9 ASVs above the breakpoint: *Faecalibacterium* (ASV45), *Sutterella* (ASV607), *Coprobacter* (ASV531), *Bacteroides* (ASV81), *Anaerostipes* (ASV364), *Ruminoccocaceae_unclassified* (ASV203), *Lactobacillus* (ASV65), *Clostridium_sensu_stricto_1* (ASV118), and *Ruminococcus_1* (ASV59). Notably, ASVs beyond the breakpoint line are largely the bacterial species responsible for the stratification of gut microbiota in samples such as *Faecalibacterium*, *Bacteroides*, and *Ruminococcus_unclassified*. We next calculated the diagnostic accuracy of the RF model by plotting the receiver operating characteristics curves (ROCs) for the above 9 taxa, MMSE, and CDR separately and in combination for each cohort group (Fig. 9B). The ROC values for these selected nine taxa were moderately accurate (area under the receiver operating characteristic curve [AUROC], 63%) (Fig. 9B), but when we included MMSE and/or CDR, we found that the RF model robustly classifies all three groups (groupwise AUROC range of 0.74 to 1.0) (Fig. 9B).

### Association of taxa with clinical parameters.

We used multivariate association with linear models (MaAsLin2) to assess the association between individual taxa and clinical metadata, including patients’ drugs (*q* ≤ 0.25). This analysis showed that *Roseburia*, *Lactobacillus*, and *Fusicatenibacter* were negatively associated with AD (see Fig. S5 in the supplemental material). Of the medication categories, there are several taxa found to be positively associated with antidepression drugs and statins. *Blautia*, *Caprococcus*, *Butyricoccus*, *Dorea*, *Lachnospiraceae* family members, and some members of *Ruminoclostridium* and *Ruminococaceae*, known to be butyrate producers, are all positively associated with antidepression drugs. Unexpectedly, we found that several taxa were significantly associated with statin medication, and of these taxa, Streptococcus and unclassified members of *Erysipelotrichaceae* were highly significantly associated with statin medication. We also observed the following taxa positively associated with statin medication: unclassified members of *Ruminococaceae* and *Lachnospiraceae*, *Phascolarctobacterium*, *Desulfovibrio*, *Caprobacter*, *Bifidobacterium*, *Butyricoccus*, *Blautia*, and *Barnesiella.*

10.1128/msystems.00004-22.5 FIG S5 Associations of the patient drugs with genus-level features. The heat map shows per-feature testing in MaAsLin 2 using linear mixed models to identify microbial species associated with drugs used by the patients. Colors of the heat map reflect the beta coefficient for drugs and age and sex from linear mixed models in MaAsLin 2 with genus-level features as outcomes. Download FIG S5, PDF file, 0.3 MB. Copyright © 2022 Yıldırım et al. 2022 Yıldırım et al. https://creativecommons.org/licenses/by/4.0/ This content is distributed under the terms of the Creative Commons Attribution 4.0 International license.

## DISCUSSION

Effective drug therapy for AD is not in the horizon; therefore, the rational and precise modulation of gut microbiota composition using diet and by other means appears to be a viable alternative in the management of AD. However, stratification of the patients based on specific microbiota features, such as taxon profile, metagenomic data, and metabolite data, is essential to achieve effective microbiota-based therapy.

In this study, we have demonstrated that the gut microbiota across the AD continuum can be stratified primarily into *Prevotella* and *Bacteroides* as the dominant subgroups, and additional subgroups can also be identified using newly developed methods. Rather than focusing on a single gut microbiota stratification method, we have exercised the best practice of implementing multiple methods to compare and contrast results and have sought support from alternative analyses. Also, all four methods we employed ranked the following taxa among the top 10 bacteria contributing to separation of the groups, suggesting these taxa play significant role in the observed community structure of the gut microbiota of the patients in this study: *Escherchia*/*Shigella*, *Faecalibacterium*, *Blautia*, *Ruminococcaceae_unclassified*, *Ruminococcaceae_UCG-002*, *Lachnospiraceae_unclassified*, and *Parabacteroides*.

PAM clustering and DMM concordantly showed three distinct clusters, one of which is consistent with the recently described Bact2 group (45). The subjects in this group are likely to have aggravated dysbiosis, as manifested from the increased abundance of the opportunistic pathogens Escherichia/*Shigella* and some species of *Bacteroides* and the lower abundance of *Faecalibacterium* and other SCFA producers. Notably, LDA analysis shuffles a similar set of taxa as the number of subgroups increases, but *Bacteroides* and *Prevotella_9* are predominantly the most abundant taxa in many of these clusters. Strikingly, Escherichia/*Shigella* dominates one of the subgroups in LDA analysis, together with opportunistic members of Klebsiella and *Enterococcus*, suggesting a dysbiotic community type may be enriched in this subgroup.

The topological data analysis (TDA) we used to stratify gut microbiota in this study deserves particular attention among other types of analysis. TDA, based on the Mapper algorithm (51), represents the underlying distribution of data in a metric space by dividing the data into overlapping similar subsets according to a filter function, with local clustering on each subset, and representing the results in an undirected network. The SAFE scores we obtained following these algorithms allowed us to identify the driver species that are responsible for community structure and showed their relationship with the metadata. We employed Bray-Curtis and Jensen-Shannon to check the variation resulting from the distance metric. *Prevotella_9*, *Bacteroides*, and *Ruminoccus_unclassified* were ranked among the top 10 taxa, with high SAFE scores, albeit in a different order, suggesting TDA is robust and consistent, even with different distance metrics. In addition to these three taxa, unclassified members of, again, other taxa within the *Ruminococcaceae* and *Lachnospiraceae* families were congruent with the other three methods we tested. Interestingly, this analysis identified the *GCA-900066575* taxon (an uncultured human intestinal bacterium) as one of the subclusters, in contrast with other methods we used. This genus is taxonomically in the family of *Lachnospiraceae*, which includes members of SCFA producers (52); still, some other members were associated with metabolic diseases such as obesity (53). Indeed, another related member of this family, *GCA-900066225*, ranked among the top 10 taxa when Bray-Curtis distance was used but enriched around AD. It is therefore important to note that TDA, unlike clustering or probabilistic partitioning methods, provided fine resolution in terms of stratification of the gut microbiota composition. Conversely, TDA did not rank the Escherichia/*Shigella* subnetwork among the top 10 taxa, and neither did the ordination show a clear association with the disease. Together, the bioinformatic tools developed in the microbiome field all have their strengths and drawbacks, and therefore overlaps in bioinformatic analyses should be pursued.

Several lines of evidence showed human cohorts in microbiome studies can be phenotypically partitioned along the *Prevotella* and *Bacteroides* stratification (54–59). A recent comprehensive report (60) provided evidence that a Mediterranean diet-based intervention is associated with specific functional and taxonomic components of the gut microbiome and that its effect is a function of microbial composition. Notably, the absence of Prevotella copri in the gut microbiomes of a subgroup of participants was associated with the protective health benefits of the dietary intervention, emphasizing the premise that microbiome-informed stratified dietary intervention would be quite effective. Nevertheless, *P. copri* is ambivalently associated with both health and disease, depending on the strain and geography (61), which prompts us to further consider its role in AD.

Taxonomically, the genus *Prevotella_9* is predicted to belong to the Prevotella copri complex (62). Comparative genome analysis of the strains of the *P. copri* complex, however, shows that some strains qualify to be assigned to even a separate species of *Prevotella* due to low genomic similarities (63, 64). Some *P. copri* strains are associated with disease states such as rheumatoid arthritis (65), while some other strains are associated with habitual diet and lifestyle (55) and are underrepresented in Westernized populations. Thus, strain-level resolution of *Prevotella_9* is needed to draw inferences. Expectedly, multiple strains of *P. copri* are likely to be part of the bacterial community in the samples. Even though we found *Prevotella_9* to be associated with the control group, the enrichment analysis using Songbird ranked some ASVs belong to *Prevotella_9* (at the species level) at the top and a few other ASVs at the bottom of the log ratio differentials, suggesting analysis beyond the species taxonomic hierarchy would provide better resolution in terms of their associations with human phenotypes. Oligotypes of these two genera in an earlier work were found to be differentially associated with a plant-based diet, and some others were associated with an animal-based diet (56). A recent report provided evidence that Bacteroides cellulosilyticus predicted weight gain more precisely than the ratio by genus of *Prevotella* to *Bacteroides*. Together, our differential enrichment analysis results are in line with these reports that species- or even strain-level resolution of these two genera could provide better predictive biomarker power for diet-based intervention studies.

One limitation of our study was that although we were able control drug-induced confounding, we did not control other potential confounders, such as diet, body mass index (BMI), and stool consistency. We largely recruited cohabiting spouses as nondemented controls sharing the same diet patterns with the patients, and carnivory is rare in Turkey. We therefore did not predict diet can strongly impact our results.

In conclusion, we demonstrate in this study that the gut microbiota along the Alzheimer’s disease continuum comprises a stratified community structure marked primarily by *Prevotella* and *Bacteroides*, but also subnetworks of other taxa exist in the community. The signature taxa when used together with MMSE and CDR can robustly classify heterogeneous groups, hence posing a potential biomarker value. This study adds to the limited number of clinical studies profiling the gut microbiota of AD continuum patients.

## MATERIALS AND METHODS

### Subject recruitment and study design.

The Istanbul Medipol University and Erciyes University Ethical Review Boards approved this study (approval no. 186/16.4.2015 and 85/20.02.2015, respectively). All participants were informed of the objectives of this study and signed a written consent form prior to their participation. The diagnoses of dementia and MCI due to AD were based on the criteria of the National Institute on Aging-Alzheimer's Association Workgroups on diagnostic guidelines for Alzheimer's disease (66, 67). Exclusion criteria for this study included history of substance abuse, any significant neurologic disease, and major psychiatric disorders, including major depression. Also, individuals were excluded who used commercial probiotics or antibiotics during the study period or within 1 month prior to providing a stool sample or who had undergone major gastrointestinal (GI) tract surgery in past 5 years. Both health centers followed the same protocols in recruiting cohorts and used kits from the same manufacturers to minimize the variations in wet lab procedures.

### Lumbar puncture and CSF biomarker assays.

Cerebrospinal fluid (CSF) samples were included in the analyses from a subset of AD patients if the patient was requested to donate a CSF sample as part of the clinically mandated diagnostic protocol. CSF samples were collected in the morning after overnight fasting using spinal needles (22 gauge) and syringes at the L3/4 or L4/5 interspace. CSF was then aliquoted into 0.5-mL nonadsorbing polypropylene tubes and stored at −80°C until assay. Biomarker molecules in CSF (Aβ_42_, phosphorylated tau (p-tau), and the p-tau/Aβ42 ratio) were measured consistent with the Alzheimer’s Association flowchart for lumbar puncture and CSF sample processing, and the biomarker levels were determined as previously described (68). Single 96-well enzyme-linked immunosorbent assay (ELISA) kits (Innogenetics, Ghent, Belgium) were used in quantitation. The cutoff values discriminating AD from controls were determined as reported before (69).

### Sample collection and DNA extraction.

Stool samples from all participants were collected in the neurology clinics of the university training hospitals. The participants were given a collection kit included a sterile tube and provided a brief instruction for collection. Self-collected samples were placed within approximately 30 min of collection in −80 freezers and kept frozen until DNA extraction.

16S rRNA gene sequencing and PCR were performed as previously described (70), with minor modifications. Briefly, genomic DNA was extracted from 220-mg fecal samples using the QiaAmp DNA stool minikit (Qiagen, Germany) per the manufacturer's instructions, with the addition of bead beating (0.1-mm zirconium beads) and lysozyme and RNase A incubation steps.

### PCR and amplicon sequencing.

To amplify the variable V3-V4 regions of the 16S rRNA gene, the primers 341 F (5′-CCTACGGGNGGCWGCAG-3′) and 805 R (5′-GACTACHVGGGTATCTAATCC-3′) were used. MiSeq sequencing adaptor sequences were added to the 5′ ends of forward and reverse primers. Approximately 12.5 ng of purified DNA from each sample was used as a template for PCR amplification in a 25-μL reaction mixture by using 2× KAPA HiFi Hot Start ready mix (Kapa Biosystems, MA, USA). For PCR amplification, the following conditions were followed: denaturation at 95°C for 3 min, followed by 25 cycles of denaturation at 95°C for 30 s, annealing at 55°C for 30 s, and extension at 72°C for 30 s, with a final extension at 72°C for 5 min. Amplified PCR products were purified with the Agencourt AMPure XP purification system (Beckman Coulter), and Nextera PCR was performed by using sample-specific barcodes. The constructed Nextera libraries were then sequenced by the Illumina MiSeq platform using MiSeq reagent kit v2 chemistry.

### Sequence processing and taxonomic assignment.

The paired-end 16S rRNA reads were first used cutadapt v1.9 program (71) for the process of quality filtering and trimming and uploaded on the DADA2 pipeline (35) integrated into the Nephele platform (72) (v2.0; http://nephele.niaid.nih.gov). Chimeric sequences are automatically removed by this pipeline, which generates both rarefied and unrarefied ASV abundance tables. We used the rarefied (10,769 reads/sample) ASV table in most downstream analysis due to large differences between some total sample reads, except for the scale-invariant DEICODE and Songbird. We removed any sequences that were classified as either being originated from *Eukarya*, *Archaea*, mitochondria, chloroplasts, or unknown kingdoms.

### Quality control.

We included no-sample DNA extractions and no-template negative-control samples in every sequencing library prepared. Using reads in the negative-control samples as a reference, we identified and removed probable contaminant reads of 13 ASVs from the ASV table, as predicted by the Decontam R package (73) using the “prevalence” method. In this method, the binary coded features across samples are compared to the prevalence in negative controls to identify contaminants. Also, we sequenced the same amplicon of an AD sample three times to check the sequencing variation. Although both centers used same protocols and kits from the same manufacturer in sequencing, we sequenced amplicons amplified from two same genomic DNA templates again from AD samples at both centers to check the center-to-center sequencing concordance. No differences could be identified between the taxonomic compositions of the samples sequenced at both centers nor between the technical replicates (PCoA, PERMANOVA, *P* = 0.1).

### Numerical ecology and statistical analysis.

Most numerical downstream analyses of ASV abundances were performed in the R environment (74). All *P* values, where appropriate, were adjusted for multiple testing using the Benjamini-Hochberg (false-discovery rate [FDR]) method. We measured within-sample microbial diversity (alpha diversity) using observed richness, Chao1, Shannon, and inverse Simpson in phyloseq (75) and tested using Kruskal-Wallis. To identify differentially abundant bacterial species, we employed the animalculus (59) and limma (76) R packages. We assessed microbial diversity between samples (beta diversity) by using multiple distance metrics, including Bray-Curtis, Jaccard, and canonical analysis of principal components (CAP). CAP analysis and the similarity percentages breakdown (SIMPER) procedure were performed using PRIMER v7 (77). Additionally, due to the compositional nature of the data, we also included robust Aitchison PCA, using the qiime2 DEICODE plugin (37) to calculate beta diversity with feature loadings. The resulting ordination was visualized using Emperor (78). We tested significance of beta diversity among groups using, again, qiime diversity plugin PERMANOVA.

Next, we used Songbird (38) for multinomial regression to rank species association with disease status with the following parameters: (formula: “MMSE+CDR+Sex+Edu+C(Group, Diff, levels=('C','MCI','AD'), –p-epochs 10000 –p-differential-prior 0.5 –p-summary-interval 1 –p-random-seed 3 –min-sample-count 1000 –min-feature-count 0). Of note, the formula structure follows Patsy formatting (https://patsy.readthedocs.io/en/latest/), such that Groups (C, MCI, AD) represent levels=[“healthy”, “mild”, “severe”] states, respectively. A null model was generated using the same parameters. The fitted model demonstrated better fit compared to the null model (pseudo Q^2^ = 0.874027). Taxa ranks were visualized using Qurro (39). Significance was determined using a Welch’s *t* test between groups, performed by GraphPad Prism.

To identify microbial species associated with the clinical metadata, including patients’ medication, we performed multivariate association with linear models (MaAsLin2) (79). The control group was excluded from this analysis, as the members of this group were not normally prescribed these medications. We employed the R package MaAsLin 2.1.0 to perform per-feature tests. We log transformed relative abundances of microbial species and standardized continuous variables into Z-scores and binary encoded medication information before including them in the MaAsLin models (*q* < 0.25 for significance).

### Stratification of gut microbiota.

We employed clustering, probabilistic partitioning, and topological data analysis approaches for the stratification of gut microbiota in the samples. The partitioning around the medoid (PAM) approach (40) clusters samples by iteratively updating each cluster’s medoid. We assigned samples to community types using the function pam() in the R package “cluster” based on Bray Curtis and Jensen Shannon distances. The number of clusters was determined by Gap statistic evaluation. Departing from the clustering approach, we next used two distinct probabilistic methods to partition the microbiota landscape, namely, Dirichlet multinomial mixture (DMM) models (41) and latent Dirichlet allocation (LDA) (42, 43). Genus-level abundances were fitted to DMM models to partition microbial community profiles into a finite number of clusters, using the Laplace approximation as previously described (41, 80).

As a second probabilistic partitioning, we performed LDA, which is a multilevel hierarchical Bayesian model (42) otherwise used for collections of discrete data, such as text corpus analysis in linguistics. LDA is a generalization of Dirichlet multinomial mixture modeling in which biological samples are allowed to have fractional membership and distinct microbial communities have different microbial signatures. Thus, for each taxon, there is a vector of probabilities across all clusters that it can be assigned to. Each cluster, therefore, has a different probability of containing taxa, indicating the chance of microbes in a particular subgroup (strata) co-occurring due to community assembly dynamics. To fit the model, we used Gibb’s sampling with the R package MetaTopics (v1.0) (81). The relative abundances of a genus collapsed table with abundances of more than 0.1% and 5% sample prevalence were input to the model. We plotted perplexity measure and log-likelihood values to estimate model performance and the optimal number of topics (subgroups of microbial assemblages) using 5-fold cross-validation. However, we observed that both parameters continued to improve with increasing subgroup number without a clear optimum, except the first jump in perplexity was near 10 topics. We therefore picked the first 10 topics for the sake of interpretability.

The final method we applied was topological data analysis (TDA) based on the Mapper algorithm (51) and network representation for stratification and association of study of high dimensional microbiome data, all integrated into the tmap tool (44). The framework enables us to reveal the association of taxa or metadata within the entire network and to identify enrichment subnetworks of different association patterns. Conceptually, the Mapper algorithm transforms a distance matrix and represents the shape of the data cloud in an undirected network. A node in the network represents a group of samples with similar microbiome profiles, and if common samples between nodes are shared, then the nodes are linked. Next, a modified special analysis of functional enrichment (SAFE) algorithm maps the metadata and taxa into the network. Finally, vectors of SAFE scores can be used in ordination to rank the driver taxa and their relationship with the metadata.

### Signature taxa.

To identify a microbial signature of severity of cognitive impairment in the AD continuum, we implemented a machine learning procedure. We first took advantage of the songbird tool to select features, including the covariates and healthy (control) and disease states (AD+MCI) in the model formula. We subsequently fit the list of ASVs selected this way into random forest models. We plotted the area under the receiver operating characteristic curve (AUROC) to show prediction performance of the models. To create the classifiers, a random forest constituted of 500 trees was computed using the default settings of the “randomForest” function implemented in the randomForest R package (v4.6-7). Mean decrease in Gini values were averaged for each ASV among the 100 random forest replicates. The ASVs with the first 20 highest mean decrease in Gini values were plotted. ASVs with mean decrease in Gini values above the breakpoint curve were chosen to be part of the classifier. Breakpoints were estimated using the “breakpoints” function included in the strucchange R package (52). We subsequently fit the list of ASVs selected this way with or without psychometric test values (i.e., MMSE and CDR) into random forest models and bootstrapped for 100 times. We plotted the area under the receiver operating characteristic curve (AUROC) to show prediction performance of the models.

### Data availability.

The 16S rRNA sequences generated by this study have been submitted to the NCBI BioProject database (https://www.ncbi.nlm.nih.gov/bioproject/) under accession no. PRJNA734525.
