# Supplementary material for: Stratification of the Gut Microbiota Composition Landscape across the Alzheimer's Disease Continuum in a Turkish Cohort
Source: mSystems. 2022 Feb 8;7(1):e00004-22. doi: 10.1128/msystems.00004-22 (PMC8823292; doi:10.1128/msystems.00004-22)
Supplement: TABLE S1 [file msystems.00004-22-st001.docx]

**Supplementary Table S1**. Levels of CerebroSpinal Fluid Biomarkers of a Subset of AD Patients

|  | Beta amiloid | fosfo tau | Total tau | Ab1–42/p-tau | Ab1–42/T-tau | Ptau/tau |
| --- | --- | --- | --- | --- | --- | --- |
| AH4 | 146 | 69 | 400 | 2.1 | 0.4 | 0.17 |
| AH5 | 1049 | 146 | 762 | 7.2 | 1.4 | 0.19 |
| AH6 | 358 | 133 | 891 | 2.7 | 0.4 | 0.15 |
| AH21 | 505 | 94 | 679 | 5.4 | 0.7 | 0.14 |
| AH22 | 410 | 65 | 467 | 6.3 | 0.8 | 0.14 |
| AH23 | 661 | 81 | 514 | 8.2 | 1.3 | 0.16 |
| AH24 | 224 | 104 | 708 | 2.2 | 0.3 | 0.15 |
| AH25 | 762 | 87 | 603 | 8.7 | 1.3 | 0.14 |
| AH26 | 318 | 64 | 451 | 4.9 | 0.7 | 0.14 |
| AH27 | 1068 | 65 | 382 | 16.4 | 2.8 | 0.17 |
| AH28 | 352 | 60 | 442 | 5.8 | 0.8 | 0.13 |
| AH29 | 384 | 78 | 596 | 4.9 | 0.6 | 0.13 |
| AH30 | 245 | 39 | 267 | 6.3 | 0.9 | 0.15 |
| AH15 | 273 | 107 | 799 | 2.5 | 0.3 | 0.13 |
