# Supplementary figures and images for: Stratification of the Gut Microbiota Composition Landscape across the Alzheimer's Disease Continuum in a Turkish Cohort
Source: mSystems. 2022 Feb 8;7(1):e00004-22. doi: 10.1128/msystems.00004-22 (PMC8823292; doi:10.1128/msystems.00004-22)

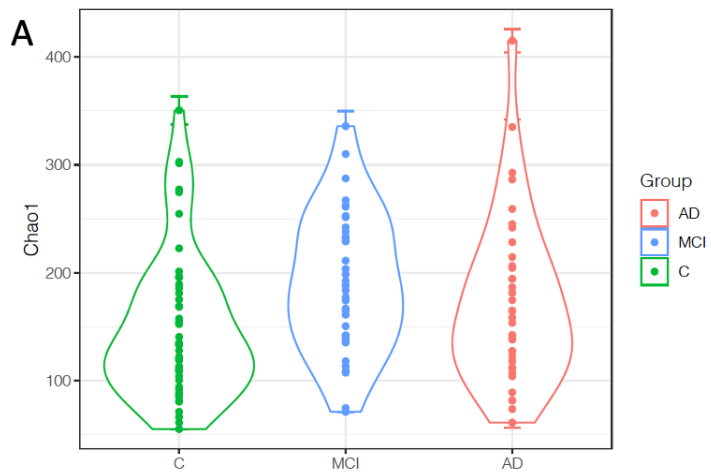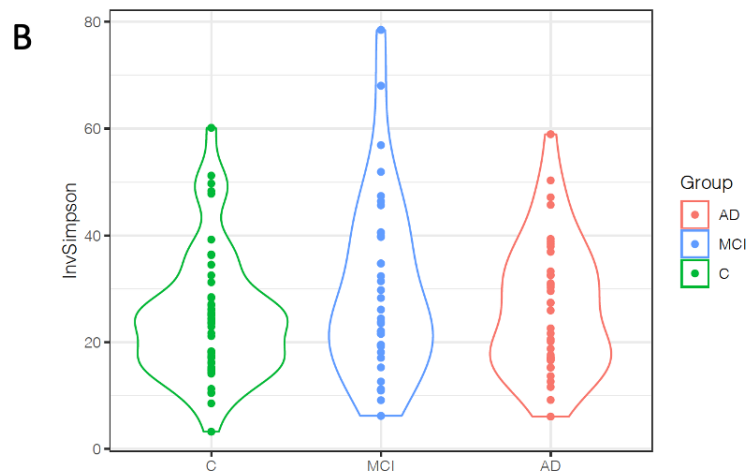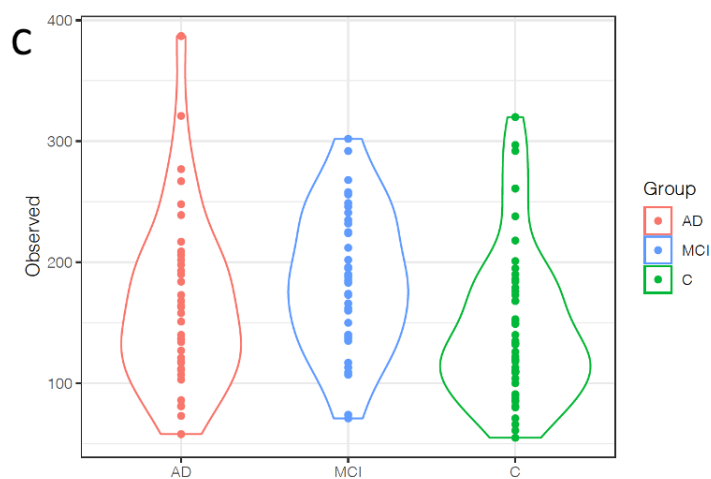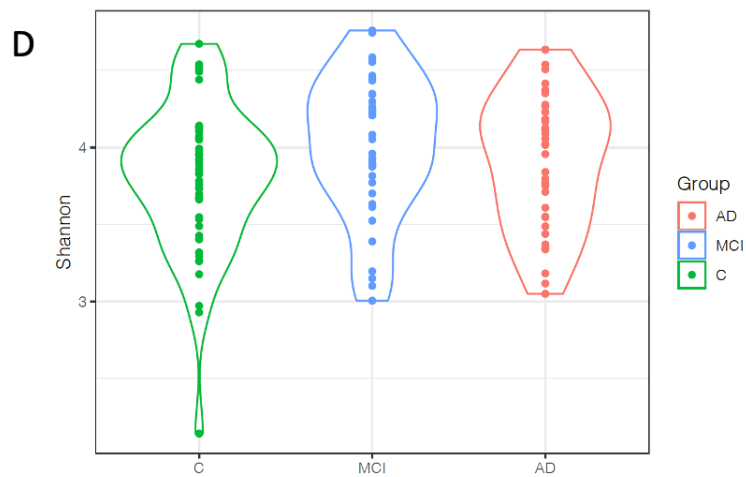

Supplement: FIG S1 [file msystems.00004-22-sf001.pdf]

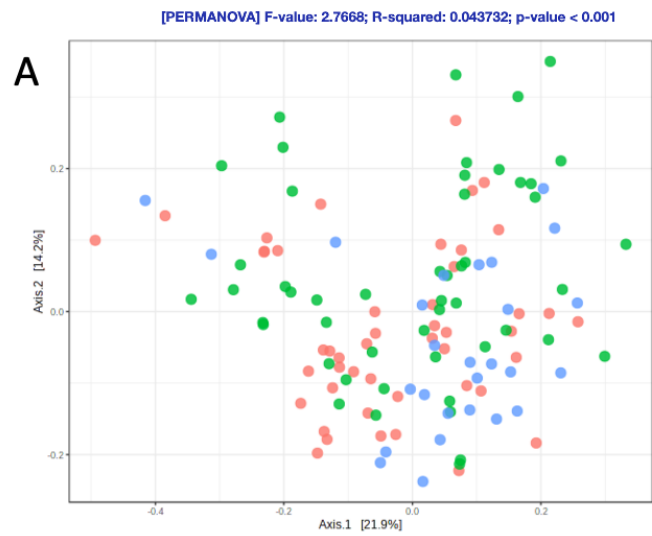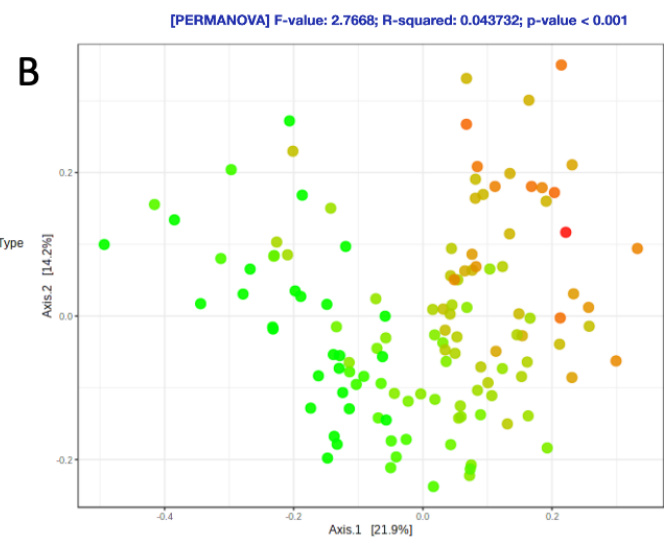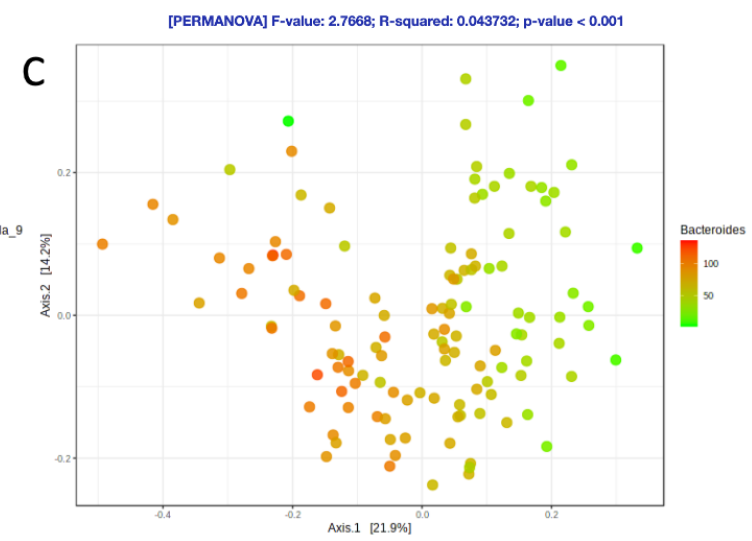

Supplement: FIG S2 [file msystems.00004-22-sf002.pdf]

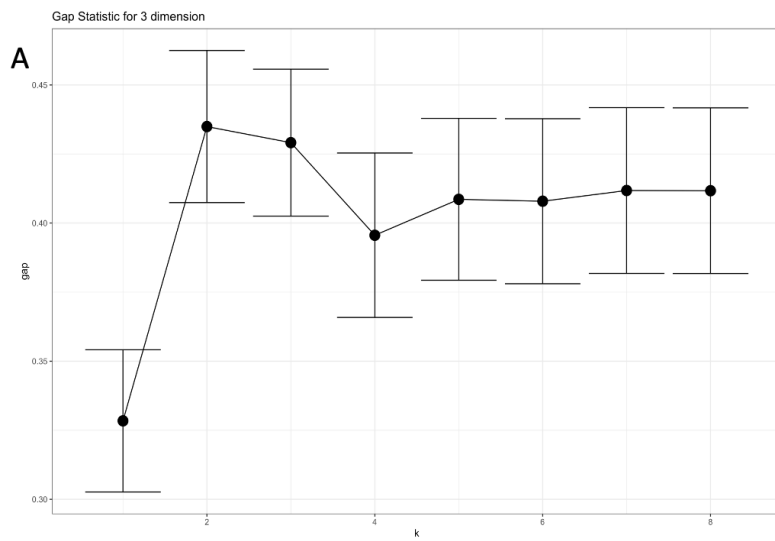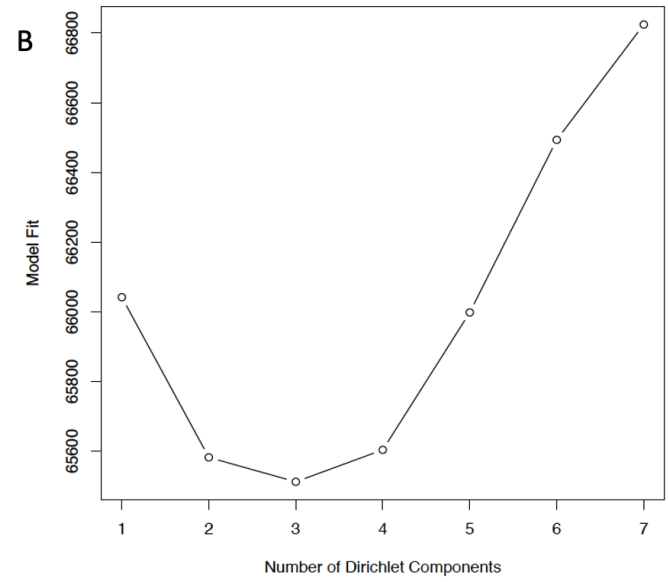

Supplement: FIG S3 [file msystems.00004-22-sf003.pdf]

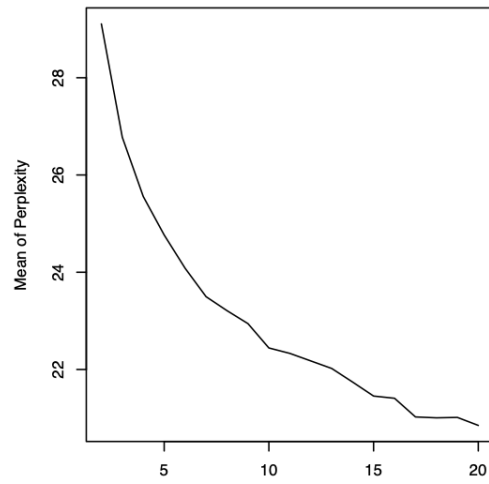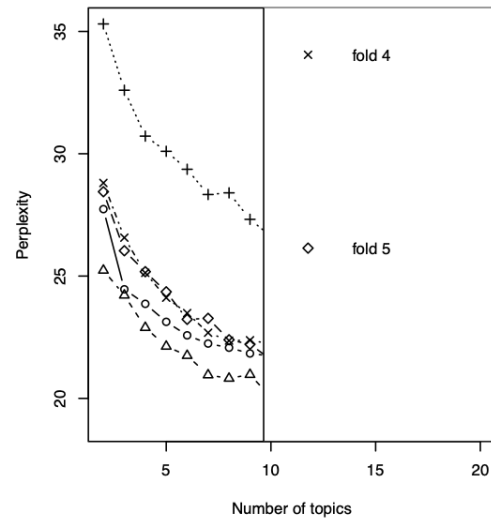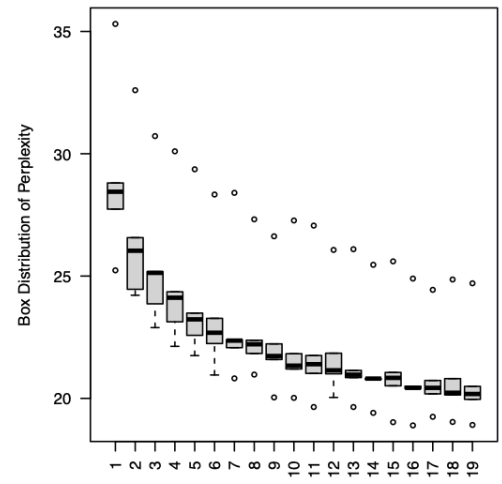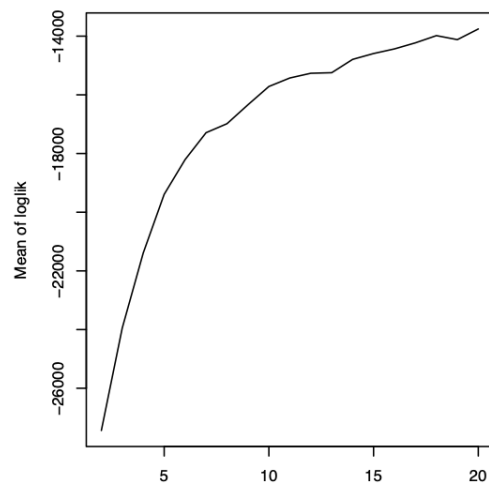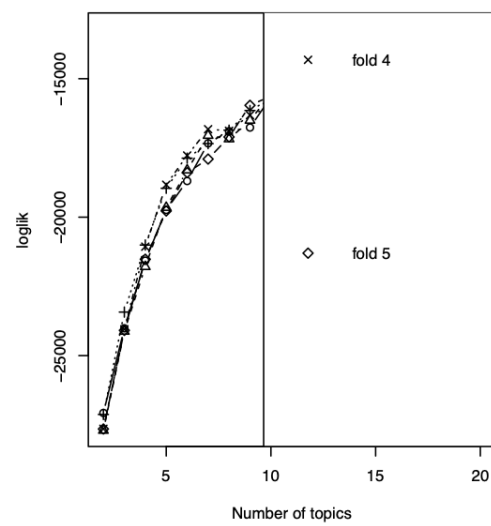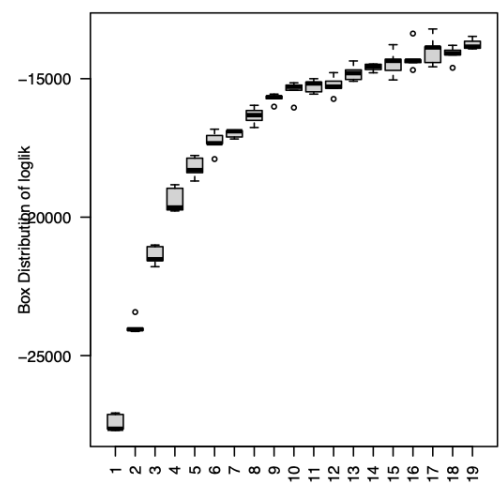

Supplement: FIG S4 [file msystems.00004-22-sf004.pdf]

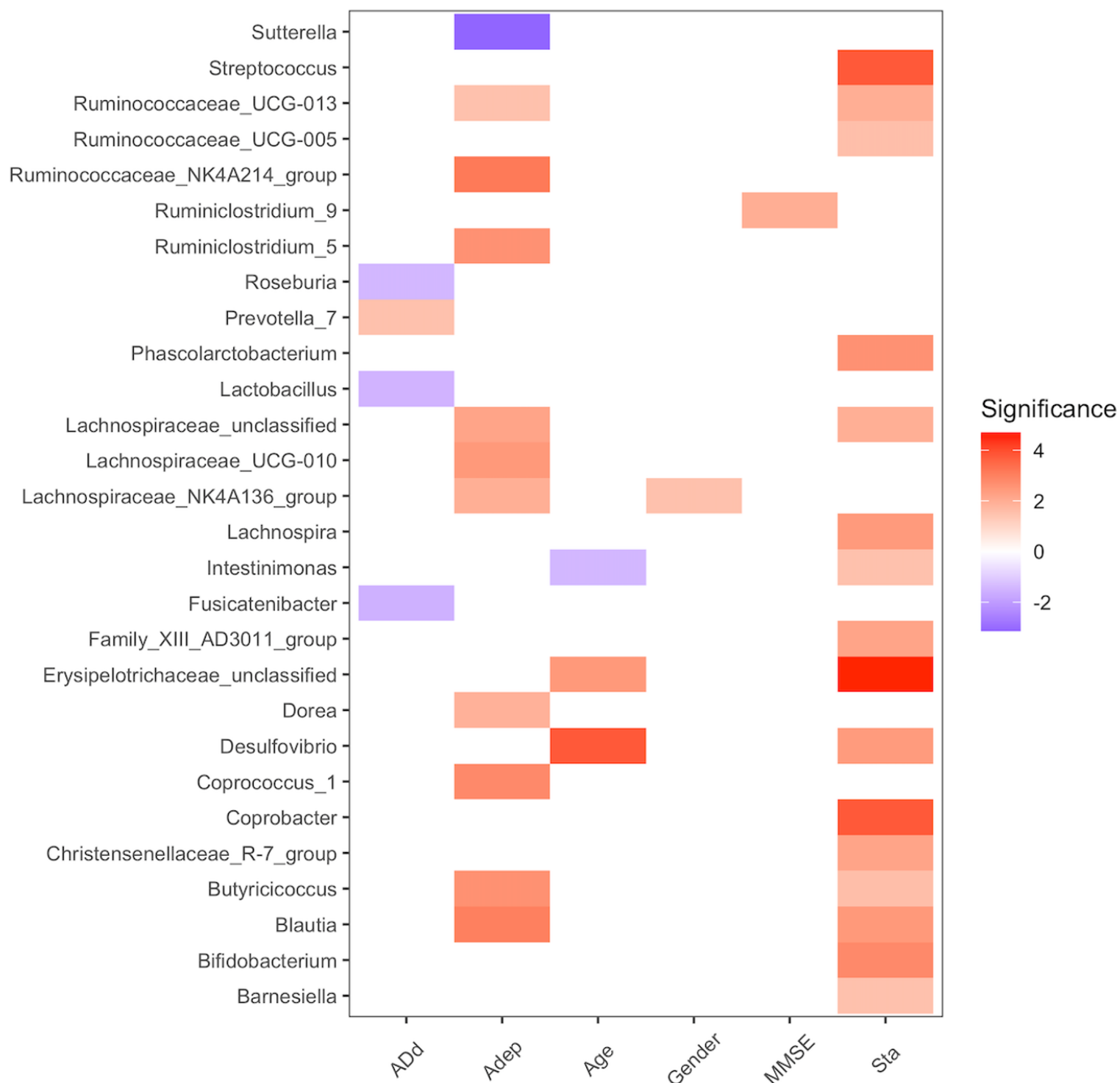

Supplement: FIG S5 [file msystems.00004-22-sf005.pdf]
