# Supplementary material for: Stratification of the Gut Microbiota Composition Landscape across the Alzheimer's Disease Continuum in a Turkish Cohort
Source: mSystems. 2022 Feb 8;7(1):e00004-22. doi: 10.1128/msystems.00004-22 (PMC8823292; doi:10.1128/msystems.00004-22)
Supplement: TABLE S3 [file msystems.00004-22-st003.docx]

**Supplementary Table S3.** PERMANOVA analysis of the clinical covariates using adonis function in Qiime2-diversity plugin.

|  | Df | SumsOfSqs | MeanSqs | F.Model | R2 | Pr(>F) |  |
| --- | --- | --- | --- | --- | --- | --- | --- |
| Group | 2 | 49.15265786 | 24.57632893 | 2.882201363 | 0.04248151 | 1.00E-04 | *** |
| Age | 1 | 19.57020153 | 19.57020153 | 2.295105249 | 0.016914074 | 0.0108 | ** |
| Sex | 1 | 21.8094682 | 21.8094682 | 2.557716377 | 0.018849421 | 0.0036 | ** |
| Edu | 1 | 13.85467788 | 13.85467788 | 1.624814332 | 0.011974279 | 0.096 | . |
| CDR | 1 | 9.096345363 | 9.096345363 | 1.066778488 | 0.007861762 | 0.3874 |  |
| MMSE | 1 | 12.61882694 | 12.61882694 | 1.47987929 | 0.010906161 | 0.1468 |  |
| DMMgroups | 1 | 33.75929878 | 33.75929878 | 3.959138781 | 0.029177384 | 1.00E-04 | *** |
| Group:Age | 2 | 20.85081073 | 10.42540536 | 1.222644669 | 0.018020875 | 0.2321 |  |
| Group:Sex | 2 | 11.22931265 | 5.614656324 | 0.658461651 | 0.009705236 | 0.8639 |  |
| Age:Sex | 1 | 8.518530172 | 8.518530172 | 0.99901492 | 0.007362369 | 0.4398 |  |
| Group:Edu | 2 | 21.35825176 | 10.67912588 | 1.252399871 | 0.018459445 | 0.208 |  |
| Age:Edu | 1 | 7.027291173 | 7.027291173 | 0.824129115 | 0.006073526 | 0.6032 |  |
| Sex:Edu | 1 | 7.821763639 | 7.821763639 | 0.917301273 | 0.00676017 | 0.5189 |  |
| Group:Age:Sex | 2 | 21.48263586 | 10.74131793 | 1.259693475 | 0.018566947 | 0.2025 |  |
| Group:Age:Edu | 2 | 27.37220439 | 13.6861022 | 1.605044534 | 0.023657166 | 0.0468 | . |
| Group:Sex:Edu | 2 | 20.32002137 | 10.16001069 | 1.191520375 | 0.017562126 | 0.2573 |  |
| Age:Sex:Edu | 1 | 5.894957388 | 5.894957388 | 0.691334099 | 0.005094876 | 0.7259 |  |
| Group:Age:Sex:Edu | 2 | 9.660118739 | 4.83005937 | 0.566447647 | 0.008349018 | 0.931 |  |
| Residuals | 98 | 835.6391285 | 8.526929882 | NA | 0.722223652 | NA |  |
| Total | 124 | 1157.036503 | NA | NA | 1 | NA |  |
|  | ***Signif. Codes* : 0 ‘***’ 0.001 ‘**’ 0.01 ‘*’ 0.05 ‘.’ 0.1 ‘ ‘ 1** | | | | | | |
|  |  | | | | | | |
